# Muscle moment arms and sensitivity analysis of a mouse hindlimb musculoskeletal model

**DOI:** 10.1111/joa.12461

**Published:** 2016-05-12

**Authors:** James P. Charles, Ornella Cappellari, Andrew J. Spence, Dominic J. Wells, John R. Hutchinson

**Affiliations:** ^1^ Neuromuscular Diseases GroupComparative Biomedical SciencesRoyal Veterinary CollegeLondonUK; ^2^ Structure and Motion LabComparative Biomedical SciencesRoyal Veterinary CollegeHatfieldUK; ^3^ Department of BioengineeringCollege of EngineeringTemple UniversityPhiladelphiaPAUSA

**Keywords:** biomechanics, muscle architecture, muscle force, musculoskeletal anatomy, rodent

## Abstract

Musculoskeletal modelling has become a valuable tool with which to understand how neural, muscular, skeletal and other tissues are integrated to produce movement. Most musculoskeletal modelling work has to date focused on humans or their close relatives, with few examples of quadrupedal animal limb models. A musculoskeletal model of the mouse hindlimb could have broad utility for questions in medicine, genetics, locomotion and neuroscience. This is due to this species’ position as a premier model of human disease, having an array of genetic tools for manipulation of the animal *in vivo*, and being a small quadruped, a category for which few models exist. Here, the methods used to develop the first three‐dimensional (3D) model of a mouse hindlimb and pelvis are described. The model, which represents bones, joints and 39 musculotendon units, was created through a combination of previously gathered muscle architecture data from microdissections, contrast‐enhanced micro‐computed tomography (CT) scanning and digital segmentation. The model allowed muscle moment arms as well as muscle forces to be estimated for each musculotendon unit throughout a range of joint rotations. Moment arm analysis supported the reliability of musculotendon unit placement within the model, and comparison to a previously published rat hindlimb model further supported the model's reliability. A sensitivity analysis performed on both the force‐generating parameters and muscle's attachment points of the model indicated that the maximal isometric muscle moment is generally most sensitive to changes in either tendon slack length or the coordinates of insertion, although the degree to which the moment is affected depends on several factors. This model represents the first step in the creation of a fully dynamic 3D computer model of the mouse hindlimb and pelvis that has application to neuromuscular disease, comparative biomechanics and the neuromechanical basis of movement. Capturing the morphology and dynamics of the limb, it enables future dissection of the complex interactions between the nervous and musculoskeletal systems as well as the environment.

## Introduction

Mice (*Mus musculus*) are currently among the most common laboratory animals used in research into vertebrate locomotor behaviour, particularly in studies into various states of neuromuscular disease or injury progression (Mancuso et al. 2011; Mathur et al. 2011; Ohri et al. 2013; Aartsma‐Rus & Van Putten, 2014; Delavar et al. 2014; Brault et al. 2015), or the sensory mechanics underlying locomotor control (Nakanishi & Whelan, 2012; Akay et al. 2014). The hindlimbs of mice are of particular interest for numerous reasons. It has been shown that there are many close muscle analogues (or even homologues) between the mouse hindlimb and the human lower limb (Burkholder et al. 1994; Delaurier et al. 2008), meaning any treatments might be extrapolated to potential patients. Furthermore, given that mice are close to the ancestral mammalian morphological condition (O'Leary et al. 2013), discerning how locomotion is controlled in the hindlimb of these non‐cursorial rodents could allow insights into how this and other hindlimb functions evolved within the mammalian lineage.

Despite the value of understanding how the hindlimbs of mice function, exactly how various motor tasks are controlled within the context of the mouse hindlimb has not been heavily studied. Locomotion and other behaviours, such as jumping and scratching, involve complex interactions between the muscular, skeletal and nervous systems, and discerning the role each of these systems plays in the initiation and maintenance of such behaviours is difficult in a purely experimental context. Furthermore, while the tools for genetically targeting and subsequently manipulating specific motor or sensory pathways are in their infancy (Deisseroth, 2011), they are expanding (Llewellyn et al. 2010; Iyer & Delp, 2014; Iyer et al. 2014), and are most powerful in mice, making a computational model of a mouse hindlimb model a further important contribution, which is the goal of this study.

Musculoskeletal modelling is a highly useful tool that can be used to dissect the contributions of each of these systems to locomotion (Delp et al. 1990; Delp & Loan, 1995, 2000; Pearson et al. 2006; Johnson et al. 2008; Arnold et al. 2010; O'Neill et al. 2013), and understanding how these interact in the mouse hindlimb has many potential benefits to human medicine. Understanding the complexities of locomotion could lead to improvements in patient rehabilitation from neuromuscular injuries, either through physiotherapy or surgical implants, or the development of treatments for various neuromuscular injuries or disorders. Furthermore, it could also aid the construction of more anatomically accurate active prosthetic limbs, many of which are in development and are currently used by amputees and those with congenital limb defects (Belić & Faisal, 2015; Hasson & Manczurowsky, 2015; Zuniga et al. 2015).

The majority of musculoskeletal models developed have focused mainly on the human body (Delp et al. 1990; Arnold et al. 2010), their close relatives (Ogihara et al. 2009; O'Neill et al. 2013) or fossil hominins (Nagano et al. 2005). A few studies have developed limb models of quadrupedal animals, such as a domestic cat (Burkholder & Nichols, 2004) and a rat (Johnson et al. 2008), although as yet, no study has investigated in detail the joint kinematics and muscle moment arms of the mouse hindlimb with such models.

This paper describes the construction of a three‐dimensional (3D) musculoskeletal model of the mouse hindlimb and pelvis. Using previously measured skeletal muscle architecture data and musculoskeletal geometry from contrast‐enhanced micro‐computed tomography (CT) scanning, the model was used to provide detailed, realistic representations of muscle moment arms and the moment‐generating capabilities of mouse hindlimb muscles throughout the range of motion of the hip, knee and ankle joints. To assess the general reliability of the mouse model, muscle moment arms were compared with those of a rat hindlimb musculoskeletal model developed by Johnson et al. (2008). A sensitivity analysis was then carried out on each force‐generating parameter as well as muscle attachment points to determine the relative effects these have on the functional capabilities of muscles.

## Materials and methods

### Model construction

The construction of a three‐dimensional musculoskeletal model requires the digital representation of two major anatomical factors: musculoskeletal geometry (i.e. the attachment points and paths of action of the muscles); and muscle force‐generating properties (from skeletal muscle architecture).

### Scanning and segmentation

To determine the attachment points and paths of action of the mouse hindlimb and pelvis musculature, a disarticulated right hindlimb and pelvic girdle of a C57BL/6 mouse (female, mass 24.9 g, age 117 days) was submerged in an aqueous solution of iodine‐potassium iodide (I_2_KI, Lugols solution) for 8 days. This staining has been previously shown to greatly enhance soft tissue contrast (Metscher, 2009a,b; Herdina et al. 2010, 2015; Cox & Jeffery, 2011; Jeffery et al. 2011; Vickerton et al. 2013; Gignac & Kley, 2014; Lautenschlager et al. 2014), and allowed individual muscles of the hindlimb and pelvis, as well as bones, to be clearly observed. For further information regarding micro‐CT scanning methods and parameters, as well as reconstructed images from these scans, see Charles et al. (2016). These reconstructed images were digitally segmented in Mimics software (Materialise, Leuven, Belgium), where 3D meshes of the pelvis, femur, patella, tibia, fibula and foot bones as well as the hindlimb and pelvis musculature were created (Charles et al. 2016). The tibia and fibula of the mouse specimen were found to fuse both proximally and distally, so these were segmented here as one bone. A total of 39 muscles was segmented (for further details, see Charles et al. 2016). The bone and muscle meshes were exported as binary STLs to open source software MeshLab (http://meshlab.sourceforge.net), where the quadratic edge collapse decimation function was applied to reduce the file size of each mesh by 75%. The STLs were then imported to Autodesk Maya (http://www.autodesk.co.uk/products/maya), where muscles and bones were placed in different segments, and coordinate systems of each segment were created. This procedure followed methods established elsewhere (Hutchinson et al. 2005, 2015).

### Hindlimb segments and joints

A total of four hindlimb segments were created: pelvis, thigh, leg (tibiofibular) and foot (pedal). These segments were manually articulated to create the hip, knee and ankle joints. The rotational centres of these joints, which also act as coordinate reference frames for each segment, were based on those from a rat musculoskeletal model created by Johnson et al. (2008; Table 1).

**Table 1:** Segment coordinate system origins, and the orientation of the respective axes in relation to bones within the musculoskeletal model

Segment	Pelvis	Thigh	Leg	Pedal
Origin	Centre of acetabulum	Centre of femoral head	Midway between femoral condyles (intercondylar point)	Centre of talus
Axis orientation				
*X*	Cranial (anterior), towards dorsilateral aspect of iliac crest	Cranial (anterior)	Cranial (anterior)	Cranial (anterior)
*Y*	Dorsal	Proximal, away from mid‐point of femoral condyles (intercondylar point)	Proximal, away from mid‐point of medial and lateral malleolus (intermalleolar point)	Proximal, towards mid‐point of tibial condyles
*Z*	Lateral	Lateral	Lateral	Lateral

The rotational centre of the hip joint was placed at the centre of the femoral head, and was modelled as a ball and socket joint between the femoral head and the acetabulum of the pelvis. This joint was given three rotational degrees of freedom: flexion–extension; adduction–abduction; and medial–lateral (internal–external) rotation. The tibial condyles were manually articulated with the femoral condyles to create the knee joint, a hinge joint with one rotational degree of freedom (flexion–extension). The centre of joint rotation was placed at the midpoint between the femoral epicondyles. The patella was included as part of the leg (tibia) segment to allow translation within the patellar groove between the femoral condyles during flexion and extension of the knee joint. The pedal segment was made to include all of the tarsal, metatarsal and phalangeal bones of the mouse's foot. The leg and pedal segments were manually articulated at the respective trochlear surfaces on the tibia and the talus to form the ankle joint, which was allowed three degrees of freedom (flexion–extension, adduction–abduction and inversion–eversion). The centre of joint rotation was placed at the midpoint between the medial and lateral malleoli of the tibia and fibula, respectively. The minimum and maximum angles of rotation for each joint were measured on a skeletonised C57BL/6 mouse hindlimb (female, mass 24.9 g, age 117 days). The soft tissue was removed and ImageJ software (Schneider et al. 2012) was used to measure the maximal flexion–extension angles from three photographs, each taken at the levels of the hip, knee and ankle joints. Maximal angles of hip and ankle adduction–abduction, as well as hip medial–lateral rotation and ankle inversion–eversion were adopted from Johnson et al. (2008; Table 2).

**Table 2:** Minimum and maximum joint angles throughout the various potential movements of the hip, knee and ankle joints

	Hip			Knee	Ankle		
	Adduction–abduction	External–internal rotation	Extension–flexion	Flexion–extension	Eversion–inversion	Adduction–abduction	Dorsiflexion–plantarflexion
Minimum	−40	−10	−30	−145	−10	−30	−50
Maximum	20	30	50	−40	30	30	50

Flexion–extension angles were measured on a skeletonised mouse hindlimb (see Materials and methods), whereas angles of abduction–adduction, internal–external rotation and eversion–inversion were incorporated from Johnson et al. (2008).

**Figure 1:**
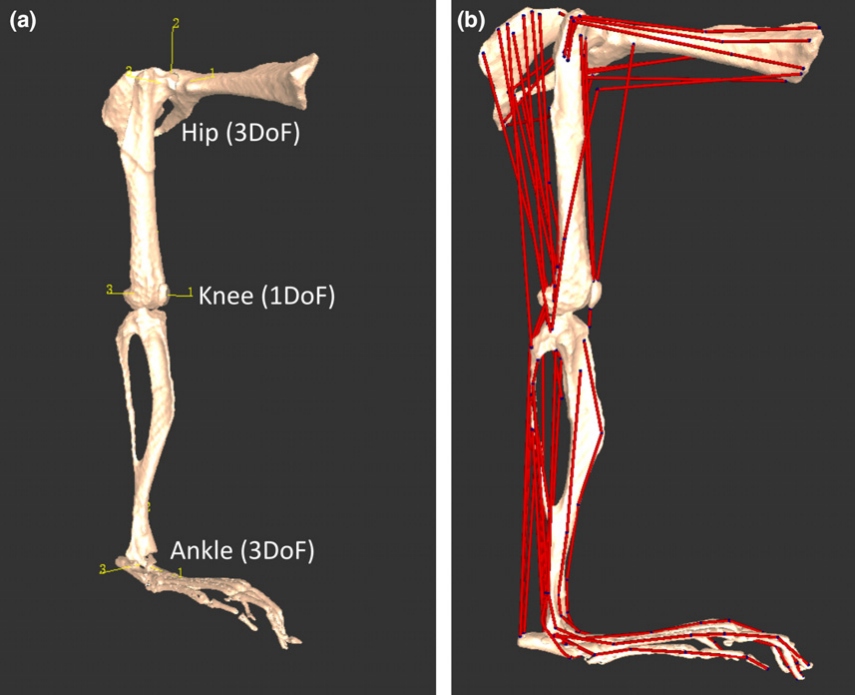
Musculoskeletal model of a mouse's right hindlimb and pelvis developed in the biomechanics software framework. Oblique craniolateral view (a) shows the rotational centres of the hip, knee and ankle joints, which were modelled with three, one and three rotational degrees of freedom, respectively. The *x*,* y* and *z* axes, labelled 1, 2 and 3, respectively, for each joint are oriented the same (at 0 ° joint angle), which is the reference position or pose. Lateral view (b) shows how the 44 musculotendon units were incorporated into the model (see Fig. 2 for more details).

Once all the segments were articulated, the axes of joint rotation were placed in the same orientation or reference position (all angles set at 0 °). The joint centres, bones and muscles were then exported to Software for Interactive Musculoskeletal Modelling software (SIMM; Musculographics, Santa Rosa, CA, USA; Delp & Loan, 1995, 2000) for construction of the musculoskeletal model (Fig. 1). The model is available for download here (https://simtk.org/home/mousehindlimb), and can be viewed and manipulated in the free OpenSim software (https://simtk.org/home/opensim).

### Muscle geometry

A total of 44 musculotendon units were used to represent the 39 muscles of the mouse hindlimb and pelvis (Charles et al. 2016) identified from micro‐CT scanning and segmentation (Tables 3, 4, 5, 6). Each musculotendon unit was placed in a functional group based on their assumed functions, considering their positions relative to joints and evidence from the literature (Burkholder et al. 1994; Lieber, 1997; Delaurier et al. 2008).

**Table 3:** Origin and insertion coordinates of 24 proximal musculotendon units included in the musculoskeletal model, derived from I_2_KI micro‐CT scanning and digital segmentation

Musculotendon unit	Abbreviation	Origin (m)				Insertion (m)			
		Segment	*x*	*y*	*z*	Segment	*x*	*y*	*z*
Gluteus (dorsal)	GM (d)	Pelvis	0.0119	0.0001	−0.0003	Thigh	−0.0012	−0.0015	0.0021
Gluteus (middle)	GM (m)	Pelvis	0.0113	−0.0090	−0.0004	Thigh	−0.0012	−0.0017	0.0021
Gluteus (ventral)	GM (v)	Pelvis	0.0111	−0.0020	0.0000	Thigh	−0.0010	−0.0019	0.0021
Obturator externus	OE	Pelvis	−0.0047	−0.0030	−0.0001	Thigh	−0.0020	−0.0010	0.0000
Obturator internus	OI	Pelvis	−0.0052	−0.0043	−0.0011	Thigh	−0.0008	0.0000	0.0003
Gemellus	GEM	Pelvis	−0.0018	0.0011	0.0001	Thigh	−0.0007	0.0004	0.0007
Quadratus femoris	QF	Pelvis	−0.0051	−0.0045	−0.0015	Thigh	−0.0019	−0.0044	0.0026
Tensor fascia latae	TFL	Pelvis	0.0110	−0.0023	−0.0003	Leg	−0.0008	−0.0019	0.0013
Adductor longus	AL	Pelvis	−0.0018	−0.0021	−0.0019	Thigh	−0.0015	−0.0135	−0.0012
Adductor magnus	AM	Pelvis	−0.0022	−0.0021	−0.0020	Thigh	−0.0017	−0.0124	0.0018
Adductor brevis	AB	Pelvis	−0.0017	−0.0020	−0.0021	Thigh	−0.0008	−0.0104	−0.0001
Gracilis posterior	GP	Pelvis	−0.0044	−0.0031	−0.0003	Leg	0.0001	−0.0047	−0.0018
Gracilis anterior	GA	Pelvis	−0.0044	−0.0029	−0.0020	Leg	0.0002	−0.0045	−0.0017
Psoas major	PMA	Pelvis	0.0104	0.0001	−0.0030	Thigh	−0.0012	−0.0022	0.0003
Psoas minor	PMI	Pelvis	0.0080	−0.0003	−0.0031	Thigh	−0.0012	−0.0020	0.0002
Iliacus	ILI	Pelvis	0.0102	−0.0024	−0.0012	Thigh	−0.0011	−0.0023	0.0004
Pectineus	PECT	Pelvis	−0.0003	−0.0018	−0.0017	Thigh	−0.0009	−0.0056	0.0006
Caudofemoralis	CF	Pelvis	−0.0026	0.0008	0.0008	Thigh	−0.0014	−0.0132	−0.0010
Semimembranosus	SM	Pelvis	−0.0041	−0.0002	0.0013	Leg	−0.0001	−0.0040	−0.0017
Semitendinosus	ST	Pelvis	−0.0057	−0.0012	0.0013	Leg	0.0001	−0.0061	−0.0017
Biceps femoris anterior	BFA	Pelvis	−0.0031	0.0009	0.0010	Thigh	−0.0019	−0.0142	0.0015
Biceps femoris posterior (cranial)	BFP (cr)	Pelvis	−0.0035	0.0007	0.0012	Leg	−0.0010	−0.0016	0.0012
Biceps femoris posterior (middle)	BFP (m)	Pelvis	−0.0043	0.0002	0.0014	Leg	−0.0019	−0.0028	0.0014
Biceps femoris posterior (caudal)	BFP (ca)	Pelvis	−0.0048	0.0000	0.0014	Leg	−0.0003	−0.0055	0.0013

These coordinates are expressed relative to the origins (0, 0, 0 as *x*,* y*,* z*) of the segment in the ‘Segment’ column, in units of metres.

**Table 4:** Origin and insertion coordinates of 20 distal musculotendon units included in the musculoskeletal model, derived from I_2_KI micro‐CT scanning and digital segmentation, in units of metres

Musculotendon unit	Abbreviation	Origin (m)				Insertion (m)			
		Segment	*x*	*y*	*z*	Segment	*x*	*y*	*z*
Rectus femoris	RF	Pelvis	0.0022	−0.0007	0.0003	Leg	0.0015	0.0007	0.0002
Vastus medialis	VM	Thigh	−0.0003	−0.0005	0.0012	Leg	0.0013	0.0008	0.0000
Vastus lateralis	VL	Thigh	−0.0004	−0.0006	0.0020	Leg	0.0013	0.0007	0.0004
Vastus intermedius	VI	Thigh	−0.0004	−0.0015	0.0015	Leg	0.0012	0.0007	0.0001
Patellar tendon	PAT	Leg	0.0011	−0.0008	−0.0001	Leg	0.0010	−0.0018	−0.0002
Popliteus	POP	Thigh	−0.0020	−0.0131	0.0012	Leg	−0.0008	−0.0030	−0.0011
Tibialis anterior	TA	Leg	0.0007	−0.0025	−0.0001	Pedal	0.0008	0.0000	−0.0006
Extensor digitorum longus	EDL	Leg	−0.0005	−0.0029	0.0016	Pedal	0.0135	−0.0019	−0.0001
Extensor hallucis longus	EHL	Leg	0.0004	−0.0042	−0.0004	Pedal	0.0093	−0.0012	−0.0012
Medial gastrocnemius	MG	Thigh	−0.0016	−0.0128	−0.0008	Pedal	−0.0021	0.0000	0.0003
Lateral gastrocnemius	LG	Thigh	−0.0019	−0.0128	0.0012	Pedal	−0.0020	0.0001	0.0007
Soleus	SOL	Leg	−0.0020	−0.0081	−0.0003	Pedal	−0.0018	0.0002	0.0005
Plantaris	PLANT	Thigh	−0.0017	−0.0125	0.0002	Pedal	−0.0020	0.0001	0.0005
Flexor digitorum longus	FDL	Leg	−0.0016	−0.0019	−0.0003	Pedal	0.0133	−0.0020	−0.0001
Tibialis posterior	TP	Leg	−0.0015	−0.0021	−0.0007	Pedal	0.0006	−0.0009	−0.0010
Peroneus longus	PL	Leg	−0.0015	−0.0035	0.0016	Pedal	0.0013	−0.0010	0.0003
Peroneus tertius	PT	Leg	−0.0019	−0.0050	0.0007	Pedal	0.0006	−0.0002	0.0010
Peroneus brevis	PB	Leg	−0.0018	−0.0088	0.0000	Pedal	0.0021	−0.0007	0.0010
Peroneus digit quarti	PDQA	Leg	−0.0020	−0.0054	0.0006	Pedal	0.0138	−0.0009	0.0007
Peroneus digiti quinti	PDQI	Leg	−0.0021	−0.0073	0.0002	Pedal	0.0116	−0.0012	0.0007

See Table 3 for further details.

**Table 5:** Force‐generating parameters of 24 proximal musculotendon units included in the musculoskeletal model

Musculotendon unit	Abbreviation	Groups	*F*_max_ (N)	*L*_f_ (m)	*L*_ts_ (m)	Pennation angle (^o^)
Gluteus (dorsal)	GM (d)	Hip rotators, hip extensors	0.936	0.01305	0.00501	20.42
Gluteus (middle)	GM (m)	Hip rotators, hip extensors	1.026	0.01271	0.00489	20.42
Gluteus (ventral)	GM (v)	Hip rotators, hip extensors	1.049	0.01242	0.00478	20.42
Obturator externus	OE	Hip rotators	0.086	0.00246	0.00096	0.00
Obturator internus	OI	Hip rotators	0.314	0.00565	0.00065	0.00
Gemellus	GEM	Hip rotators	0.179	0.00143	0.00001	0.00
Quadratus femoris	QF	Hip rotators	2.030	0.00465	0.00110	0.00
Tensor fascia latae	TFL	Hip rotators	0.000	0.00000	0.00000	0.00
Adductor magnus	AM	Hip adductors	0.614	0.00760	0.00302	0.00
Adductor longus	AL	Hip adductors	0.402	0.00745	0.00255	0.00
Adductor brevis	AB	Hip adductors	0.234	0.00642	0.00176	0.00
Gracilis posterior	GP	Hip adductors, hip extensors	0.345	0.00912	0.00435	0.00
Gracilis anterior	GA	Hip adductors, hip extensors	0.402	0.00882	0.00607	0.00
Psoas major	PMA	Hip flexors	1.338	0.00697	0.00501	15.54
Psoas minor	PMI	Hip flexors	1.088	0.00578	0.00390	12.57
Iliacus	ILI	Hip flexors	0.549	0.00857	0.00275	0.00
Pectineus	PECT	Hip flexors	0.363	0.00277	0.00181	15.18
Caudofemoralis	CF	Hip extensors	0.554	0.01137	0.00307	0.00
Semimembranosus	SM	Hip extensors, knee flexors	1.916	0.01165	0.00409	0.00
Semitendinosus	ST	Hip extensors, knee flexors	1.299	0.01111	0.00480	0.00
Biceps femoris anterior	BFA	Hip extensors, knee flexors	0.876	0.01145	0.00383	0.00
Biceps femoris posterior (cranial)	BFP (cr)	Hip extensors, knee flexors	0.725	0.01008	0.00491	0.00
Biceps femoris posterior (middle)	BFP (m)	Hip extensors, knee flexors	0.728	0.01004	0.00478	0.00
Biceps femoris posterior (caudal)	BFP (ca)	Hip extensors, knee flexors	0.611	0.01197	0.00406	0.00

Maximum force (*F*_max_), fibre length (*L*_f_) and pennation angle (^o^) were derived from previously measured skeletal muscle architecture (Charles et al. 2016). Tendon slack length (*L*_ts_) was estimated using a numerical optimisation procedure from Manal & Buchanan (2004). Grouping was based on presumed functions during locomotion (see Materials and methods).

**Table 6:** Force‐generating parameters of 20 distal musculotendon units included in the musculoskeletal model

Musculotendon unit	Abbreviation	Groups	*F*_max_ (N)	*L*_f_ (m)	*L*_ts_ (m)	Pennation angle (^o^)
Rectus femoris	RF	Knee extensors	4.162	0.00534	0.00853	15.89
Vastus medialis	VM	Knee extensors	1.098	0.00653	0.00768	16.15
Vastus lateralis	VL	Knee extensors	2.828	0.00681	0.00735	15.53
Vastus intermedius	VI	Knee extensors	0.367	0.00606	0.00702	10.92
Patellar tendon	PAT	Knee extensors	0.000	0.00000	0.00000	0.00
Popliteus	POP	Knee flexors	0.307	0.00206	0.00203	0.00
Tibialis anterior	TA	Ankle dorsiflexors	2.422	0.00490	0.01180	16.58
Extensor digitorum longus	EDL	Ankle dorsiflexors	0.368	0.00635	0.02378	12.39
Extensor hallucis longus	EHL	Ankle dorsiflexors	0.069	0.00593	0.01793	9.56
Medial gastrocnemius	MG	Ankle plantarflexors, knee flexors	1.750	0.00550	0.01395	14.24
Lateral gastrocnemius	LG	Ankle plantarflexors, knee flexors	3.784	0.00541	0.01389	17.28
Soleus	SOL	Ankle plantarflexors	0.591	0.00316	0.00740	11.43
Plantaris	PLANT	Ankle plantarflexors	0.880	0.00431	0.01517	17.10
Flexor digitorum longus	FDL	Ankle plantarflexors	1.896	0.00431	0.02761	15.20
Tibialis posterior	TP	Ankle plantarflexors	0.549	0.00359	0.01500	15.44
Peroneus longus	PL	Ankle everters, ankle plantarflexors	0.647	0.00378	0.01408	14.90
Peroneus tertius	PT	Ankle everters, ankle plantarflexors	0.457	0.00339	0.01122	12.46
Peroneus brevis	PB	Ankle everters, ankle plantarflexors	0.396	0.00229	0.01005	11.46
Peroneus digiti quarti	PDQA	Ankle everters, ankle plantarflexors	0.112	0.00393	0.02357	12.42
Peroneus digiti quinti	PDQI	Ankle everters, ankle plantarflexors	0.102	0.00362	0.01959	9.44

Details as in Table 5.

**Figure 2:**
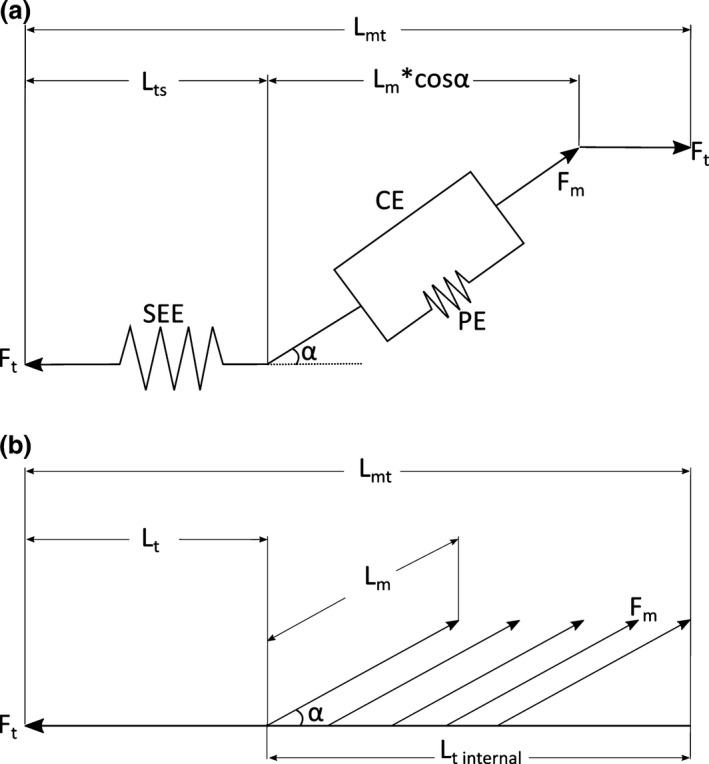
(a) A generic Hill‐type muscle model used to estimate muscle contractile dynamics in the musculoskeletal model. It consists of a contractile element (CE) connected in parallel to a passive elastic element (PE), which together represent the muscle fibres and their mechanical properties. Muscle force (*F*_m_) depends primarily on fibre length and activation level. The contractile element is in series with a series elastic element (SEE), which represents the elastic properties of the entire tendon. Tendon force (*F*_t_) is equal to *F*_m_*cos*α*, where *α* represents fibre pennation angle. The entire musculotendon unit length (*L*_mt_) is equal to *L*_t _+ *L*_m_*cos*α*, where *L*_m_ represents muscle length. Adapted from Delp et al. (1990) and O'Neill et al. (2013). (b) A realistic representation of a typical unipennate muscle, with an external tendon (*L*_t_) and a large internal tendon (*L*_t internal_), to which many muscle fibres attach. These variables together represent *L*_ts_ in the model. See Table 9 for a comparison between *L*_t_ and *L*_ts_.

Each musculotendon unit is represented in the software by a generic Hill‐type muscle model (Fig. 2a; Zajac, 1989; Delp & Loan, 1995, 2000), each of which was scaled to characterise each muscle by specifying four parameters: maximum isometric force (*F*_max_); optimal fibre length (*L*_f_); tendon slack length (*L*_ts_); and pennation angle (*θ*). These parameters were derived from averaged architecture data gathered from eight C57BL/6 mouse hindlimbs (females; body mass 23.45 ± 2.73 g, age 107.8 ± 34.86 days; mean ± SD) through dissections. These limbs were placed in 10% neutral buffered formalin for 24 h with the hip, knee and ankle held at 90 ° to maximise the potential of achieving optimal fibre length throughout fixation. The muscles were then individually dissected to determine their mass, length, fibre length, pennation angle and physiological cross‐sectional area (PCSA). As has been assumed elsewhere (O'Neill et al. 2013), optimal fibre length was taken here to be equivalent to empirically measured fibre length. For more detailed methods and full muscle architecture data, see Charles et al. (2016). These methods used here were similar to those employed in other architecture studies of rodent limbs (Burkholder et al. 1994; Delaurier et al. 2008; Eng et al. 2008; Mathewson et al. 2012). Note that these data are strain‐, sex‐, mass‐ and age‐matched to the subject used for micro‐CT scanning to determine the model's underlying geometry, a practice described in the construction of other musculoskeletal models including, but not limited to, rodents (Brown et al. 2003; Arnold et al. 2010; Johnson et al. 2011). Maximum isometric force was calculated for each muscle by multiplying muscle PCSA by force per unit area, or isometric stress (*σ*), taken here as 0.3 N mm^−2^ (Zajac, 1989; Medler, 2002; Hutchinson, 2004). *L*_ts_ is defined as the length beyond which tendons begin to develop passive elastic force. This is regarded an important parameter to accurately determine when constructing a musculoskeletal model (Delp & Zajac, 1992), however it is impossible to measure experimentally. Using optimal fibre length, minimum–maximum normalised fibre length and minimum–maximum musculotendon unit length across the maximal joint ranges of motion, *L*_ts_ was estimated for each muscle using a numerical optimisation procedure (Manal & Buchanan, 2004). It is important to note that within the Hill‐type muscle model (Fig. 2a; Zajac, 1989), the ‘tendon’ represents the entire in‐series elasticity of the muscle, which in theory includes both the external and internal (aponeurosis) portions of the tendon (Fig. 2b). Therefore, even muscles with no external tendon [e.g. M. gluteus maximus (GM) or M. semimembranosus (SM)] will have *L*_ts_ values in the musculoskeletal model. This is a common modelling assumption and has been described previously (O'Neill et al. 2013).

**Figure 3:**
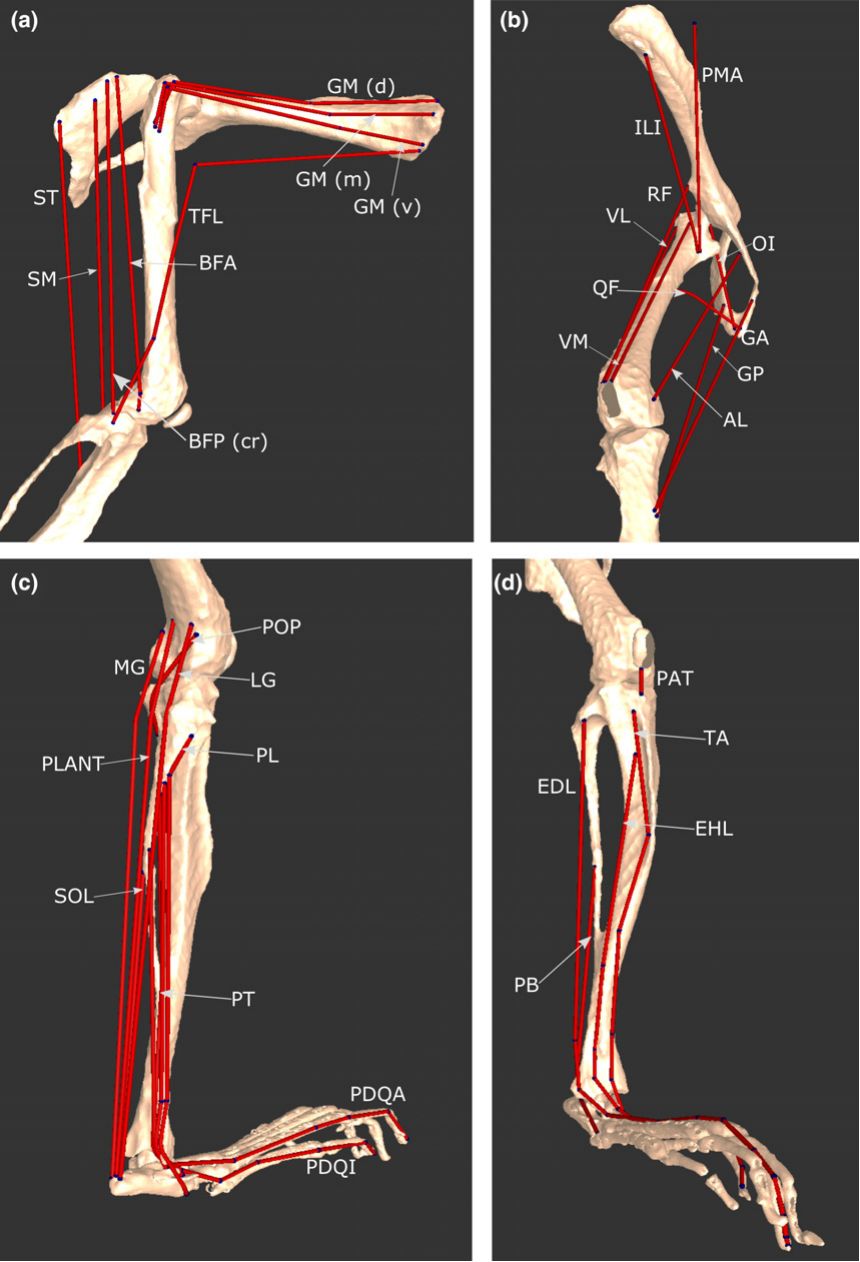
Select musculotendon units of the mouse hindlimb and pelvis musculoskeletal model. (a) Various hip extensors in a lateral view. (b) A craniomedial view, showing hip flexors, adductors and knee extensors. (c) A caudolateral view, showing ankle plantarflexors, ankle everters (except PB), as well as POP, a knee flexor. (d) A craniolateral view, showing ankle dorsiflexors, PB and PAT. For abbreviations, see Tables 3 and 4.

**Figure 4:**
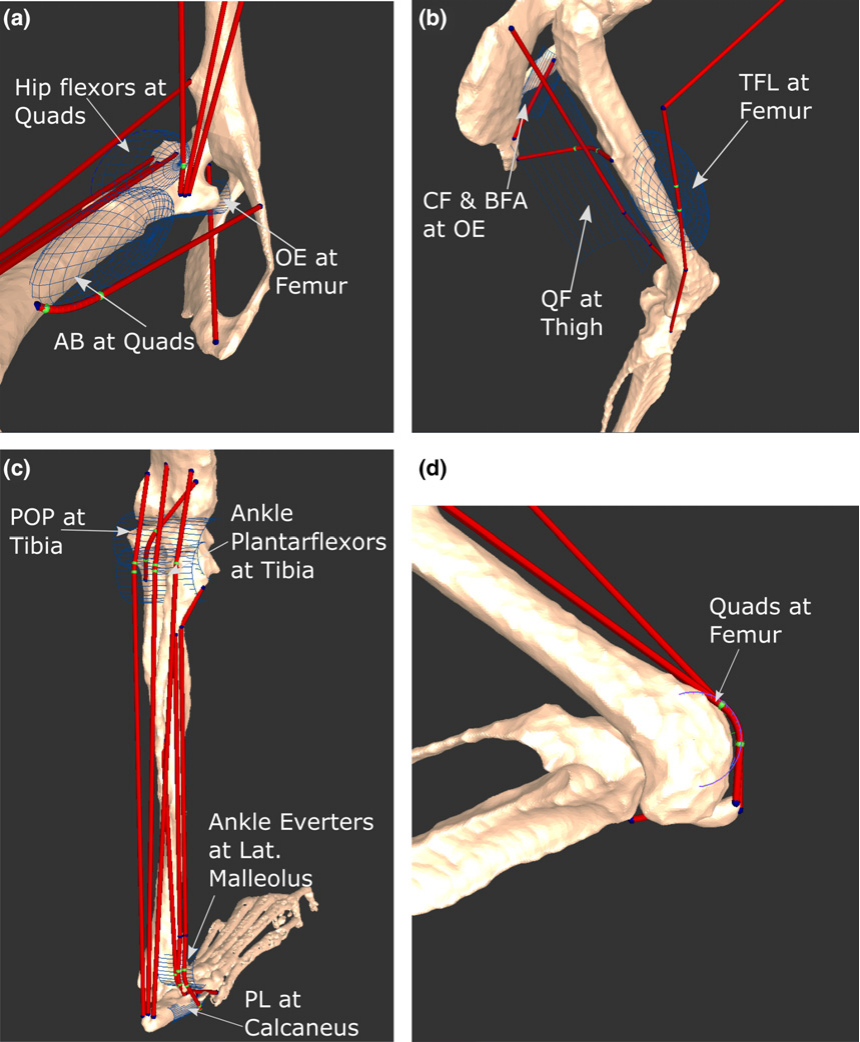
Positions and names of several wrapping objects placed into the musculoskeletal model. Depending on the anatomical landmark being modelled by the object, wrapping objects were shaped as either a semi‐torus or semi‐cylinder. Green points represent areas of the muscles that are being acted on by the wrapping object. Select wrapping objects in the medial hip (a), lateral thigh (b), posterior leg (c) and distal femoral (d) regions are shown. For muscle abbreviations, see Tables 3, 4, 5, 6.

The 3D meshes of muscles created from reconstructed micro‐CT scans were used to accurately determine the origin and insertion coordinates of the pelvic and hindlimb musculotendon units in the model, with each attachment point placed as close to the observed centroid of muscle attachment as possible (Fig. 3). There were two large muscles in which it was deemed necessary to use multiple musculotendon models to represent their paths of action. GM was represented here by three separate muscles [GM (dorsal), GM (middle) and GM (ventral)], to account for its broad origin on the iliac crest, while M. biceps femoris posterior (BFP) was also represented by three muscles [BFP (cranial), BFP (middle) and BFP (caudal)] due to its broad insertion on the lateral aspect of the fibula and adjacent fascia. This is common in musculoskeletal modelling (Delp et al. 1990; van der Helm et al. 1992; Arnold et al. 2010), as there is substantial evidence that different portions of a muscle can work heterogeneously to produce total muscle force (Gatesy & English, 1993). In cases where it was found that modelled muscles would pass through other anatomical landmarks (either bone, retinaculae or other muscles), either ‘via points’ or wrapping surfaces were used to constrain them to realistic paths of action (Fig. 4; for wrapping surface properties, see Tables 7 and 8).

**Table 7:** Properties of cylindrical wrapping objects placed into the mouse musculoskeletal model

Musculotendon unit	Segment	Location	*r*(*x*)	*r*(*y*)	*r*(*z*)	*t*(*x*)	*t*(*y*)	*t*(*z*)	Radius	Height
Gracilis anterior	Pelvis	Caudal pubic ramus	3.5400	−0.3500	−53.7800	−0.0072	−0.0029	−0.0013	0.0025	0.0050
Obturator internus	Thigh	Lesser trochanter of femur	167.2700	9.9200	−50.8400	−0.0016	−0.0014	0.0005	0.0006	0.0025
Quadratus femoris	Thigh	Caudal to proximal third of femoral shaft	85.4000	−0.5200	−124.6900	−0.0039	−0.0053	0.0017	0.0018	0.0075
Rectus femoris	Thigh	Femoral condyles	3.1900	−1.3400	−129.4700	−0.0009	−0.0137	0.0002	0.0010	0.0030
Vastus medialis	Thigh	Femoral condyles	3.1900	−1.3400	−129.4700	−0.0009	−0.0137	0.0002	0.0010	0.0030
Vastus lateralis	Thigh	Femoral condyles	3.1900	−1.3400	−129.4700	−0.0009	−0.0137	0.0002	0.0010	0.0030
Vastus intermedius	Thigh	Femoral condyles	3.1900	−1.3400	−129.4700	−0.0009	−0.0137	0.0002	0.0010	0.0030
Caudofemoralis	Thigh	Caudal aspect of greater trochanter	96.6400	−51.9300	13.5600	−0.0027	−0.0016	−0.0002	0.0005	0.0025
Biceps femoris anterior	Thigh	Caudal aspect of greater trochanter	96.6400	−51.9300	13.5600	−0.0027	−0.0016	−0.0002	0.0005	0.0025
Popliteus	Leg	Posterior aspect of tibial condyles	8.9300	−9.3100	−1.7500	−0.0007	−0.0019	0.0000	0.0010	0.0040
Medial gastrocnemius	Leg	Proximal leg and distal thigh	0.6500	2.9700	3.2100	−0.0011	−0.0029	0.0000	0.0010	0.0030
Lateral gastrocnemius	Leg	Proximal leg and distal thigh	0.6500	2.9700	3.2100	−0.0011	−0.0029	0.0000	0.0010	0.0030
Plantaris	Leg	Proximal leg and distal thigh	0.6500	2.9700	3.2100	−0.0011	−0.0029	0.0000	0.0010	0.0030
Peroneus longus	Leg	Lateral malleolus	−4.0400	4.7600	8.3300	−0.0008	−0.0177	0.0000	0.0008	0.0015
Peroneus tertius	Leg	Lateral malleolus	−4.0400	4.7600	8.3300	−0.0008	−0.0177	0.0000	0.0008	0.0015
Peroneus brevis	Leg	Lateral malleolus	−4.0400	4.7600	8.3300	−0.0008	−0.0177	0.0000	0.0008	0.0015
Peroneus digiti quarti	Leg	Lateral malleolus	−4.0400	4.7600	8.3300	−0.0008	−0.0177	0.0000	0.0008	0.0015
Peroneus digiti quinti	Leg	Lateral malleolus	−4.0400	4.7600	8.3300	−0.0008	−0.0177	0.0000	0.0008	0.0015
Peroneus longus	Pedal	Lateral aspect of tarsal bones	80.3700	81.3700	41.8100	−0.0003	−0.0003	0.0009	0.0003	0.0021

‘*r*’ notes the rotation (in ^o^) of the object about the *x*,* y* and *z* segment axes. ‘*t*’ notes the translation (in units of m) of the objects from the segment origins (see also Hutchinson et al. 2005, 2015).

**Table 8:** Properties of spherical and elliptical wrapping objects placed into the mouse musculoskeletal model

Musculotendon unit	Segment	Location	*r*(*x*)	*r*(*y*)	*r*(*z*)	*t*(*x*)	*t*(*y*)	*t*(*z*)	Radius (*x*)	Radius (*y*)	Radius (*z*)
Obturator externus	Pelvis	Body of ischium	−14.8700	−24.5900	51.7300	−0.0039	−0.0010	0.0000	0.0025	0.0005	0.0015
Caudofemoralis	Pelvis	Body of ischium	98.7500	−40.8600	141.7200	−0.0043	−0.0010	0.0053	0.0015	0.0005	0.0025
Biceps femoris anterior	Pelvis	Body of ischium	98.7500	−40.8600	141.7200	−0.0043	−0.0010	0.0053	0.0015	0.0005	0.0025
Semitendinosus	Pelvis	Caudal pubic ramus near pubic tubercle	0.0000	0.0000	0.0000	−0.0057	−0.0018	0.0006	0.0006	0.0006	0.0006
Quadratus femoris	Thigh	Proximal third mid femoral shaft	4.6800	10.0800	3.8500	−0.0011	−0.0054	0.0014	0.0009	0.0040	0.0012
Psoas major	Thigh	Proximal femoral shaft	3.5600	−2.0100	0.6900	−0.0001	−0.0027	0.0016	0.0010	0.0030	0.0015
Psoas minor	Thigh	Proximal femoral shaft	3.5600	−2.0100	0.6900	−0.0001	−0.0027	0.0016	0.0010	0.0030	0.0015
Iliacus	Thigh	Proximal femoral shaft	3.5600	−2.0100	0.6900	−0.0001	−0.0027	0.0016	0.0010	0.0030	0.0015
Pectineus	Thigh	Mid‐medial femoral shaft	−0.4800	1.0900	0.0900	−0.0004	−0.0047	0.0006	0.0006	0.0017	0.0008
Adductor brevis	Thigh	Mid‐medial femoral shaft	5.0900	−2.0300	2.8200	−0.0008	−0.0070	0.0001	0.0010	0.0035	0.0010
Tensor fascia latae	Thigh	Mid‐lateral femoral shaft	9.4900	0.7900	1.3900	−0.0004	−0.0078	0.0014	0.0015	0.0035	0.0013
Caudofemoralis	Thigh	Caudal aspect of proximal femur	115.2000	−18.2100	−101.1700	−0.0010	−0.0008	0.0010	0.0018	0.0010	0.0015
Biceps femoris anterior	Thigh	Caudal aspect of proximal femur	115.2000	−18.2100	−101.1700	−0.0010	−0.0008	0.0010	0.0018	0.0010	0.0015
Caudofemoralis	Thigh	Caudal aspect of mid femoral shaft	−91.7200	−3.1300	−87.4300	−0.0019	−0.0055	0.0015	0.0015	0.0005	0.0030
Biceps femoris anterior	Thigh	Caudal aspect of mid femoral shaft	−91.7200	−3.1300	−87.4300	−0.0019	−0.0055	0.0015	0.0015	0.0005	0.0030
Gracilis posterior	Leg	Proximal tibia	−175.9800	−1.3400	172.5900	−0.0007	−0.0064	0.0002	0.0025	0.0060	0.0020
Semimembranosus	Leg	Proximal tibia	−175.9800	−1.3400	172.5900	−0.0007	−0.0064	0.0002	0.0025	0.0060	0.0020
Semitendinosus	Leg	Proximal tibia	−175.9800	−1.3400	172.5900	−0.0007	−0.0064	0.0002	0.0025	0.0060	0.0020
Biceps femoris posterior (caudal)	Leg	Lateral aspect, proximal third of leg	10.7200	11.0000	−4.7500	−0.0013	−0.0057	−0.0005	0.0012	0.0038	0.0020

‘*r*’ notes the rotation (in ^o^) of the object about the *x*,* y* and *z* segment axes. ‘*t*’ notes the translation (in units of m) of the objects from the segment origins.

### Model analysis

#### Muscle moment arms

Once the muscles were positioned in the model and checked for consistently realistic motions throughout all degrees of freedom, muscle moment arms were plotted as a function of joint angle for each muscle of the hindlimb and pelvis. Moment arm plots can be used to test the reliability of the muscle attachment point, ‘via point’ or wrapping object placement, as well as gain a further understanding of each muscle's function throughout the joint angles used in limb function. As a further test of the robustness of the muscle placement within the mouse hindlimb model, the moment arms were compared with those from a previously developed hindlimb musculoskeletal model of another non‐cursorial rodent, a rat (Johnson et al. 2008). Due to the differences in body size and shape between the mouse and rat, absolute moment arm values could not be compared. Instead, these values were scaled based on the respective segment lengths of the mouse or rat model, giving dimensionless values that could be reasonably compared between the two species.

#### Sensitivity analysis

The output of the musculoskeletal model can be represented as the isometric moment (rotational force or torque; in units of Nmm) that can be produced by a muscle throughout the range of motion of any respective joint, which is a function of both its force‐generating properties and its musculoskeletal geometry. A sensitivity analysis was carried out to determine the relative effect of changing muscle force‐generating parameters or geometry on the model output. The analysis involved altering, in turn, *F*_max_, *L*_f_, *L*_ts_ and pennation angle of a particular muscle by +5% or +1 standard deviation of the mean (the latter provided in Supplementary Information) while holding other parameters constant, as well as moving the coordinates of its origin and insertion ± 0.5 mm along a defined axis.

Six musculotendon units were tested; GM (dorsal) (GM d), M. psoas major (PMA), M. semitendinosus (ST), M. rectus femoris (RF), M. tibialis anterior (TA), and M. lateral gastrocnemius (LG). When changing attachment points, the origin of GM (d) was moved ventrally or dorsally along the lateral surface of the iliac crest, while the insertion was moved proximally or distally along the lateral aspect of the proximal femur. The origin of PMA was moved cranially or caudally along the lumbar vertebrae, and the insertion was moved proximally or distally along the lesser trochanter of the femur. The origin of ST was moved cranially or caudally along the ischial tuberosity of the pelvis, and the insertion was moved proximally or distally along the medial aspect of the tibia. The origin of RF was moved cranially or caudally along the anterior inferior iliac spine of the pelvis, and the insertion was moved cranially or caudally along the base of the patella. The origins of TA and LG were moved proximally or distally along the leg, while the insertions were moved cranially or caudally along the medial cuneiform and the calcaneus bones of the foot, respectively.

## Results

### Muscle moment arms

**Figure 5:**
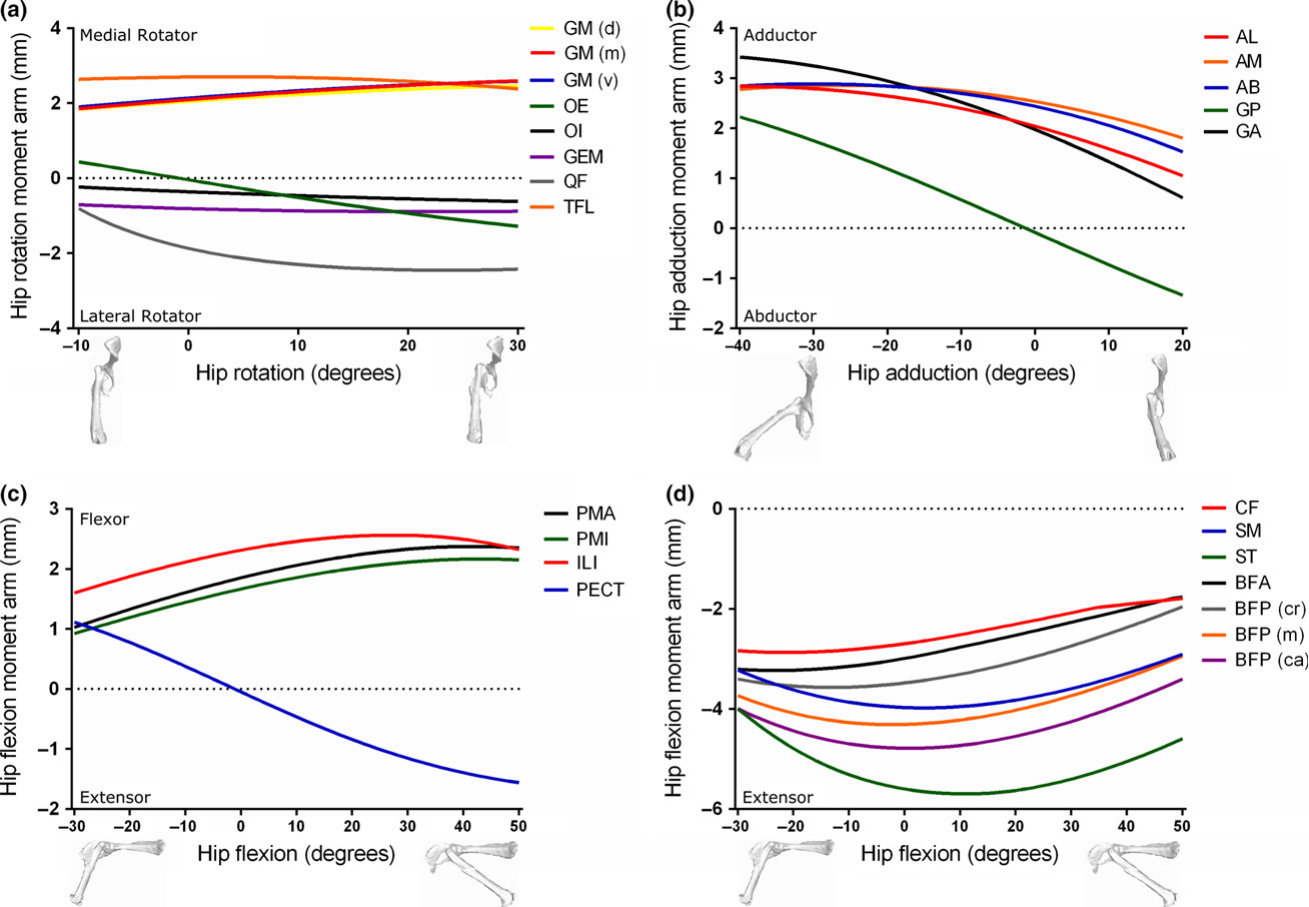
Moment arms of musculotendon units acting around the hip joint through medial–lateral rotation (a), adduction–abduction (b) and flexion–extension (c and d) in the mouse musculoskeletal model.

**Figure 6:**
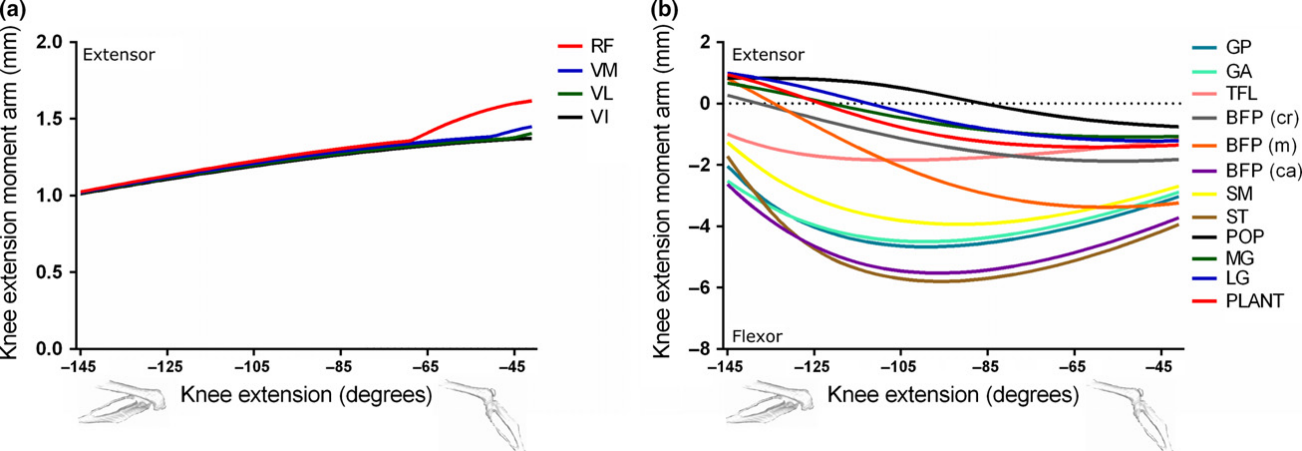
Moment arms of musculotendon units acting around the knee joint through extension–flexion (a and b) in the mouse musculoskeletal model.

**Figure 7:**
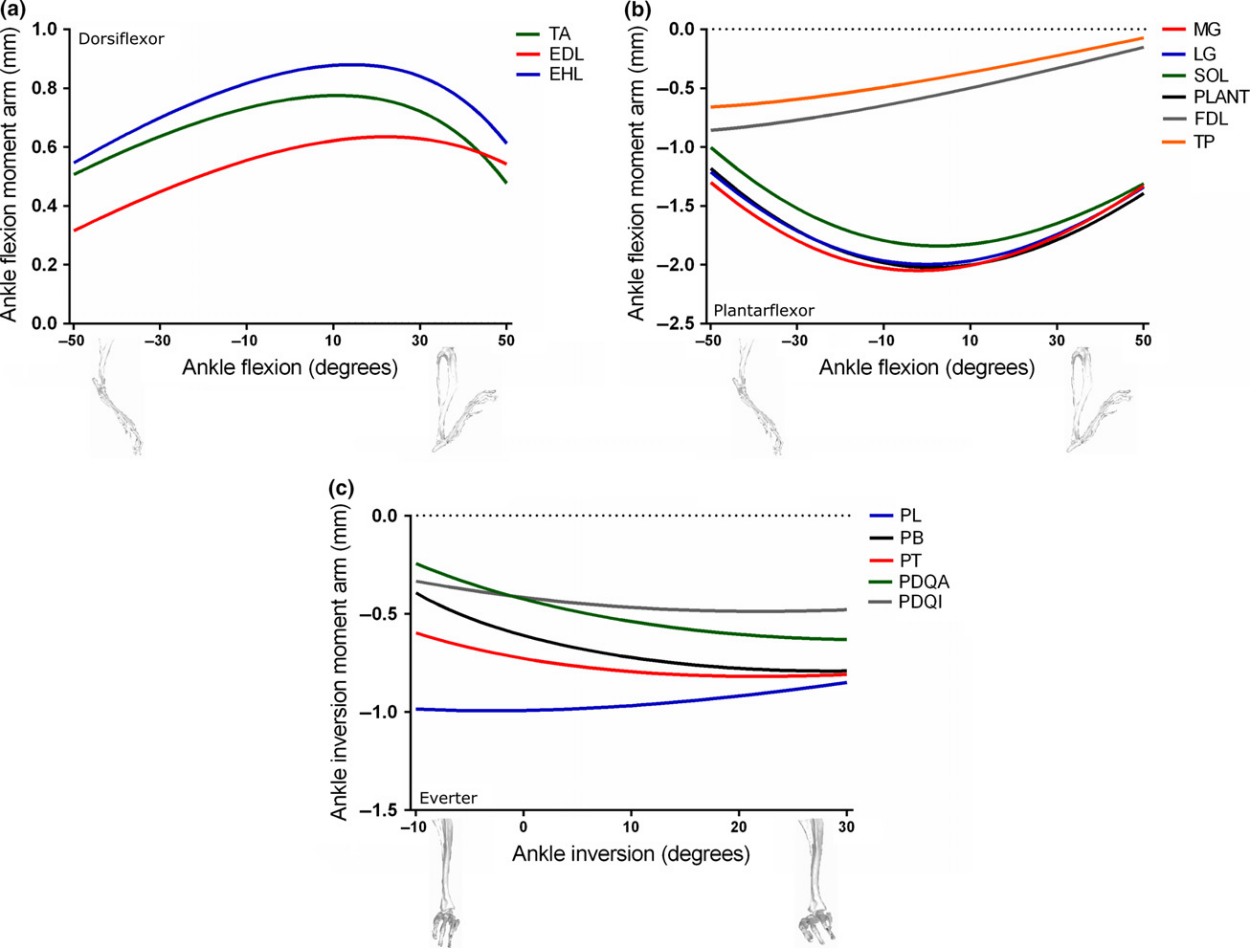
Moment arms of musculotendon units acting around the ankle joint through dorsiflexion–plantarflexion (a and b) and inversion–eversion (c) in the mouse musculoskeletal model.

Plots of muscle moment arm vs. joint angle for each musculotendon unit of the mouse hindlimb and pelvis model are shown in Figs 5, 6, 7. Moment arms were plotted through all the modelled rotations of each joint (Table 1). Of the hip rotators (Fig. 5a), M. tensor fascia latae (TFL) had the greatest peak medial hip rotation moment arm through the range of hip rotation (2.70 mm at 3 ^o^), while M. quadratus femoris (QF) had the greatest peak lateral rotation moment arm (−2.45 mm at 23 ^o^). The hip rotation moment arm of M. obturator externus (OE) crossed a moment arm of zero with a negative slope at about 0 ^o^ of hip rotation, meaning that it would function as a medial rotator when the hip is laterally rotated, but a lateral rotator when medially rotated. The other small hip rotators [M. obturator internus (OI), M. gemellus (GEM) and QF] also showed trends to cross zero at larger angles of lateral hip rotation (< −10 °).

Of the hip adductors (Fig. 5b), M. gracilis anterior (GA) had the greatest peak hip adduction moment arm when the hip is abducted (< 0 ^o^), with a peak value of 3.42 mm at −40 ^o^. When the hip is adducted (> 0 ^o^), M. adductor magnus (AM) had the greatest peak hip adduction moment arm (1.99 mm at 1 ^o^).

M. iliacus (ILI) had the greatest peak hip flexion moment arm of the hip flexors (2.56 mm at 27 ^o^; Fig. 5c), with PMA and M. psoas minor (PMI) showing similar trends but smaller peak values throughout the range of hip flexion. M. pectineus (PECT) hip flexion moment arm crossed zero in a negative slope at about −2 ^o^, changing from a hip flexor at angles of hip extension (< 0 ^o^) to an extensor at hip flexion (> 0 ^o^).

Of the hip extensors (Fig. 5d), ST had the greatest peak hip extension moment arm (−5.70 mm at 11 ^o^ of hip flexion). The other hip extensors [e.g. M. caudofemoralis (CF), SM, BFA and BFP] showed similarly shaped curves through hip flexion–extension, but with smaller peak values.

The muscles of the quadriceps femoris group [consisting of M. rectus femoris (RF), M. vastus medialis (VM), M. vastus lateralis (VL) and M. vastus intermedius (VI)] were evident as the major knee extensors (Fig. 6a). Due to a shared insertion onto the patella, the knee extension moment arm values of these muscles were similar throughout knee extension until about −65 ^o^, where the moment arm of RF increased and peaked at 1.62 mm at full knee extension.

Several different muscle groups can contribute to knee flexion (Fig. 6b). Of the ‘hamstring’ muscles, those that also extend the hip, ST had the highest knee flexion moment arm, with a peak of −5.80 mm at −95 ^o^. Many of the ‘triceps surae’ group [MG, LG and M. plantaris (PLANT)], which should primarily function to plantarflex the ankle, also can assist in knee flexion, although the peak knee flexion moment arm of PLANT (−1.42 mm at −61 ^o^) was much less than that of the hip extensors.

Of the muscles that can dorsiflex the ankle joint (Fig. 7a), M. extensor hallucis longus (EHL) had the greatest peak ankle flexion moment arm (0.88 mm at 14 ^o^), with TA and M. extensor digitorum longus (EDL) showing similarly shaped curves but lower peaks.

M. medial gastrocnemius (MG) had the greatest ankle plantarflexion moment arm amongst the posterior and medial compartments of the leg (Fig. 7b), with a peak of −2.05 mm at roughly neutral ankle angles. LG and PLANT had similarly shaped curves but slightly lower peaks.

Amongst the muscles that should primarily function as ankle everters, M. peroneus longus (PL) had the greatest peak ankle eversion moment arm, with a value of −0.99 mm at −3 ^o^ of eversion (Fig. 7c).

#### Comparison with rat hindlimb model

**Figure 8:**
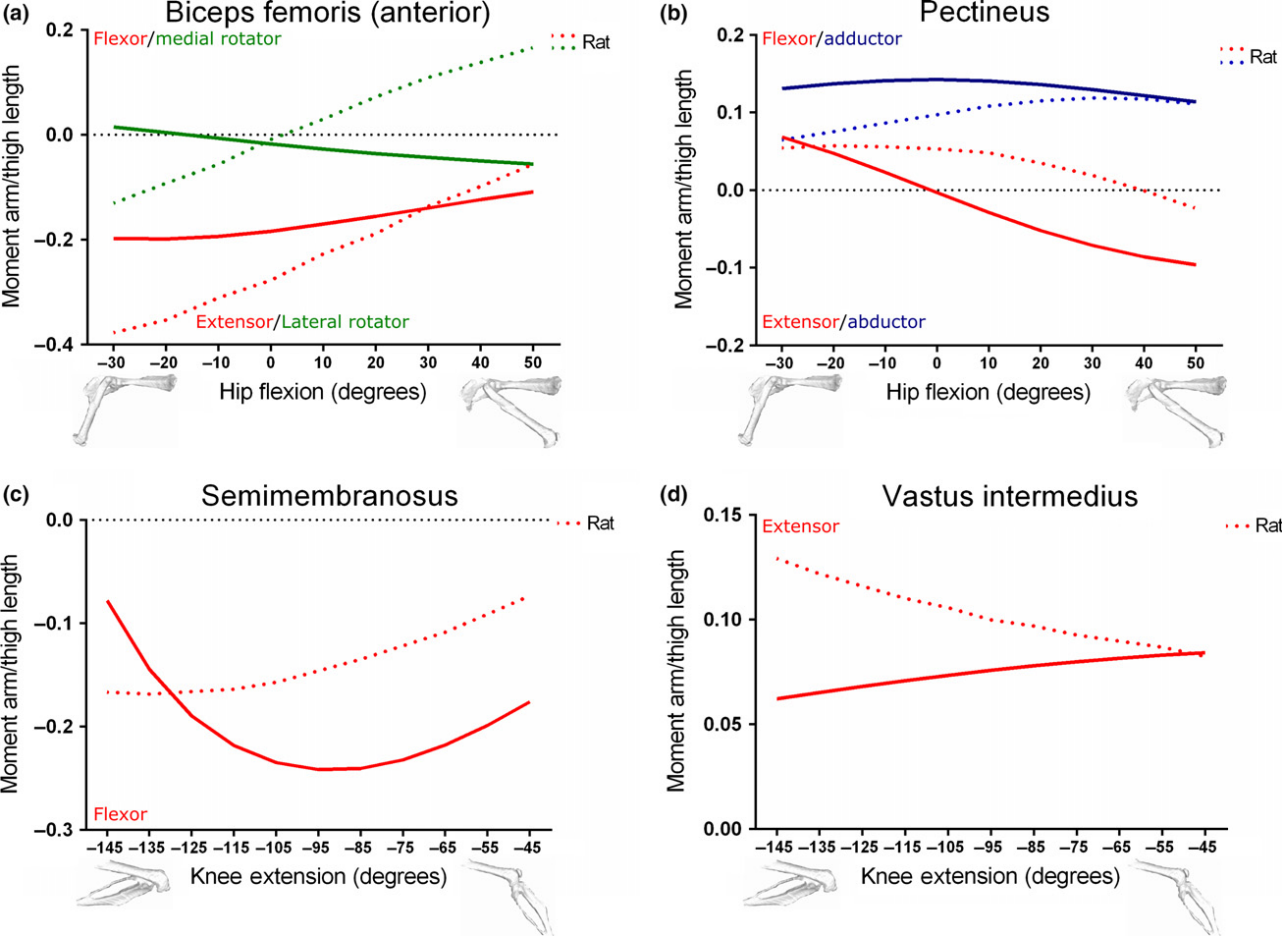
Comparison between moment arms of the Biceps femoris (anterior) (a), Pectineus (b), Semimembranosus (c) and Vastus intermedius (d) muscles within the mouse and rat (Johnson et al. 2008) hindlimb musculoskeletal models, scaled to respective thigh lengths. Mouse thigh length: 16.25 mm; rat thigh length: 35.00 mm.

**Figure 9:**
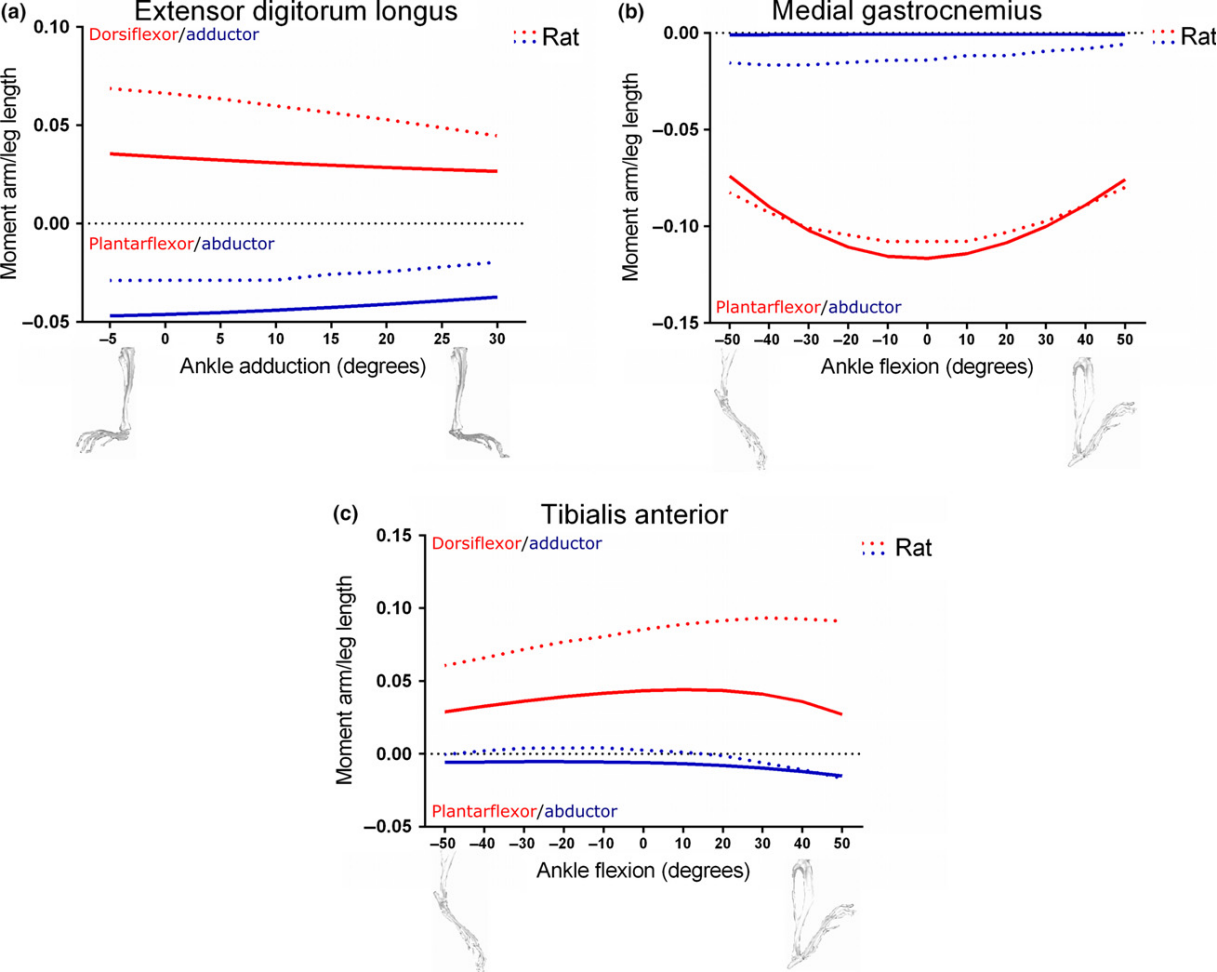
Comparison between moment arms of the Extensor digitorum longus (a), medial Gastrocnemius (b) and Tibialis anterior (c) muscles within the mouse and rat (Johnson et al. 2008) hindlimb musculoskeletal models, scaled to respective leg lengths. Mouse leg length: 17.56 mm; rat leg length: 39.57 mm.

To facilitate a comparison between this study's mouse hindlimb musculoskeletal model and that of a rat (Johnson et al. 2008), muscle moment arms of the two models were scaled to their respective segment lengths and thereby represented by dimensionless numbers (Figs 8 and 9). The thigh and leg segments of the mouse hindlimb were measured here as 16.25 mm and 17.56 mm, respectively, which are roughly half the lengths of the same segments of the rat hindlimb, as measured by Johnson et al. (2008).

Figure 8a shows the hip rotation and extension moment arms of BFA in the mouse and rat models, normalised to thigh length, plotted against hip flexion angle. At the limits of hip extension, mouse BFA showed a peak medial rotation moment arm/thigh length of 0.015, although the BFA changed to a peak lateral rotation moment arm/thigh length of −0.055 at the limits of hip flexion. The rat model's BFA showed the opposite condition, with a peak lateral rotation moment arm/thigh length of −0.13 at extreme hip extension and a medial rotation moment arm/thigh length of 0.16 at hip flexion. The hip extension moment arm vs. hip flexion angle curves of both the mouse and rat BFA were similar in shape when scaled to thigh length, but differed in terms of peak value. Mouse BFA had a peak of −0.20 at −20 ^o^ of hip extension, while rat BFA peaked at −0.38 at roughly −30 ^o^.

In terms of hip adduction moment arm/thigh length vs. hip flexion, the mouse's and rat's PECT were similar in both shape and peak (Fig. 8b), with the mouse's PECT showing a peak of 0.14 and the rat's PECT peaking at 0.12. As previously shown in Fig. 4C, the mouse's PECT had a hip flexion moment arm that crossed zero at neutral hip flexion angles (~0 ^o^). The mouse's PECT also had a peak hip flexion moment arm/thigh length of 0.069 at extreme hip extension angles, and a peak hip extension moment arm/thigh length of −0.096 at the limits of hip flexion. The rat's PECT, however, crossed zero at 40 ^o^ of hip flexion, and had a peak hip flexion moment arm/thigh length of 0.057 at −20 ^o^ and a peak extension moment arm/thigh length at 50 ° of hip flexion.

The peak hip extension moment arms of the mouse's and rat's SM were similar when normalised to thigh length (Fig. 8c), with peaks of −0.24 and −0.17, respectively. However, the angles at which these peaks occurred were different, with the mouse's SM hip extension moment arm/thigh length peaking at −95 ^o^ and the rat's SM peaking at −135 ^o^ of knee flexion.

The knee extension moment arm/thigh length vs. knee flexion angle plots for the mouse and rat VI (Fig. 8d) differed in both shape and peak. The mouse VI showed a positive slope and a peak of 0.084 at 41 ^o^, the limit of knee extension, while the rat VI had a negative slope and a peak of 0.13 at 145 ^o^.

Both the ankle dorsiflexion and abduction moment arm/leg (i.e. tibia) length vs. ankle adduction angle plots for EDL were similar in shape between the mouse and the rat models when scaled to leg length, although the peaks differed slightly (Fig. 9a). The mouse's EDL dorsiflexion moment arm/leg length peaked at 0.036, whereas abduction peaked at −0.047. The rat's EDL had a dorsiflexion peak of 0.069 and an abduction peak of −0.029. All these peaks occurred at about −5 ^o^ of ankle adduction.

The shape and peak of the mouse's and rat's MG muscle's ankle plantarflexion moment arm/leg length vs. ankle flexion were similar (Fig. 9b), with peaks of −0.12 (at 0 ^o^) and −0.11 (between 0 ^o^ and 10 ^o^), respectively. Both species were also found to have small ankle abduction moment arm/leg length peaks, with the mouse's MG having a negligible peak of −0.0009, and the rat's having a larger peak of −0.017 at 40 ^o^ of ankle plantarflexion.

The peak (normalised) ankle dorsiflexion moment arm/leg length vs. ankle flexion curve of the mouse's TA was less than half of that of the rat's, with values of 0.044 and 0.093, respectively (Fig. 9c). The mouse's TA had a small peak ankle abduction moment arm/leg length of −0.015 at the limits of ankle dorsiflexion, and this is similar in the rat's TA, which had a similar peak of −0.017. However, at ankle plantarflexion angles, the rat's TA had an ankle adduction moment arm, with a small peak of 0.0042 at 10 ^o^ when normalised to segment length.

### Sensitivity analysis

#### Changing architecture

**Figure 10:**
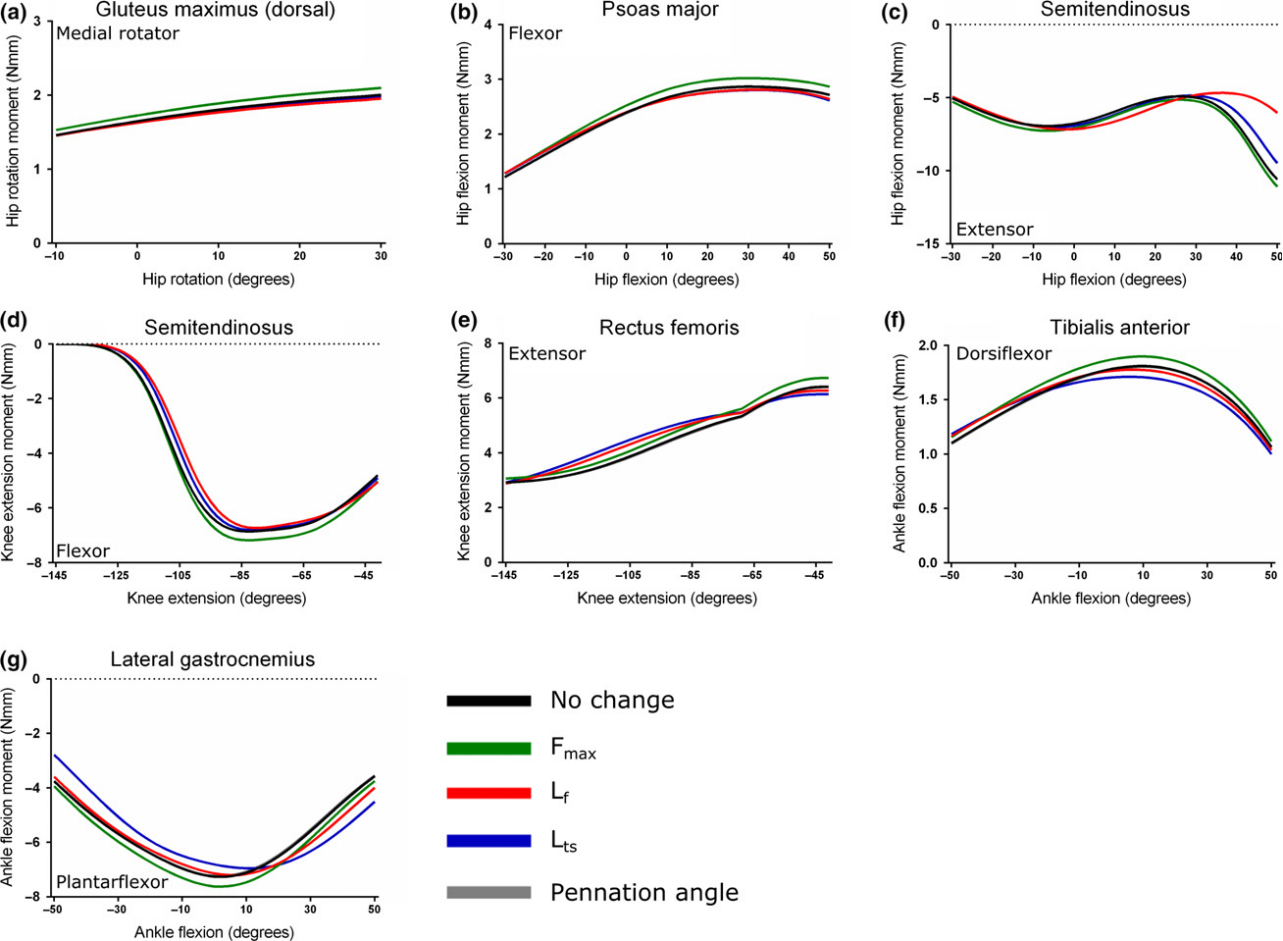
Sensitivity analysis of the Gluteus maximus (dorsal) (a), Psoas major (b), Semitendinosus (c, d), Rectus femoris (e), Tibialis anterior (f) and lateral Gastrocnemius (g) muscles. Here, maximum isometric force (*F*_max_), muscle fibre length (*L*_f_), tendon slack length (*L*_ts_) and fibre pennation angle were increased by 5% in turn to test the effect on maximal muscle moment.

For the first part of the sensitivity analysis carried out on a musculoskeletal model of a mouse's hindlimb and pelvis, the force‐generating properties of select musculotendon units were altered +5% in turn, in order to test the relative effects on maximal isometric moment vs. joint angle (Fig. 10). To test the sensitivity of the model to potential inaccuracies in the muscle architecture data, the same parameters were increased by 1 standard deviation around the mean, using the same methods mentioned above, and tested for effects on maximal isometric moment vs. joint angle (Fig. S1).

For GM (d) (Fig. 10a) and PMA (Fig. 10b), maximal hip rotation and hip flexion moment, respectively, were both insensitive to changes in any force‐generating parameter other than *F*_max_. The peak moment of GM (d) increased from 1.82 Nmm at 26 ^o^ of hip rotation, to 1.91 Nmm, while PMA's peak hip flexion moment increased from 2.87 Nmm to 3.02 Nmm at 30 ^o^ of hip flexion.

Both the hip flexion and knee extension maximal moments of ST were highly sensitive to changes in several force‐generating parameters. Increasing both *L*_f_ and *L*_ts_ had the greatest effects on the maximal hip flexion moment (Fig. 10c), causing reductions of the peak value from −10.60 Nmm at the limits of hip flexion to −6.04 Nmm (a 43% decrease) and −9.49 Nmm (an 11% decrease), respectively. Altering *L*_f_ also changed the angle at which peak maximal hip extension occurred, which was −7.17 Nmm at −3 ^o^ of hip extension, a 32% decrease.

The maximal knee extension moment of ST was relatively insensitive to changes in these parameters (Fig. 10d), with an increase in *F*_max_ causing the greatest increase in peak, from −6.86 Nmm to −7.18 Nmm at −83 ^o^ of knee flexion.

Increases in *F*_max_, *L*_ts_ and *L*_f_ all resulted in an increase in RF maximal knee extension moment (Fig. 10e), with *L*_f_ and *L*_ts_ having the greatest effects at large angles of knee flexion. However, an increase in *F*_max_ caused the largest change in peak moment, with an increase from 6.42 Nmm at −41 ^o^ of knee flexion, to 6.74 Nmm.

The TA maximal ankle dorsiflexion moment was most sensitive to changes in *F*_max_ and *L*_ts_ (Fig. 10f). Increasing *F*_max_ caused an increase in peak dorsiflexion moment, from 1.81 Nmm to 1.90 Nmm at 10 ^o^ of ankle dorsiflexion. Increasing *L*_ts_ decreased the dorsiflexion moment to 1.71 Nmm, a 6% decrease, and also slightly changed the angle of peak moment to 6 ^o^.

Similar to the TA, LG's maximal plantarflexion moment was most sensitive to changes in *F*_max_ and *L*_ts_ (Fig. 10g). Increasing *F*_max_ resulted in an increase in peak plantarflexion moment, from −7.27 Nmm to −7.62 Nmm at 2 ^o^ of ankle dorsiflexion. Increasing *L*_ts_ caused the peak moment to decrease to −6.85 Nmm (6% decrease), and also changed the angle of this peak to 10 ^o^ of ankle dorsiflexion.

For muscles with a measurable fibre pennation angle, increasing this parameter by 5% had little effect on maximal isometric moment, which follows the mathematical formulation of the model involving the cosine of this angle (Fig. 2; Zajac, 1989).

#### Changing geometry

**Figure 11:**
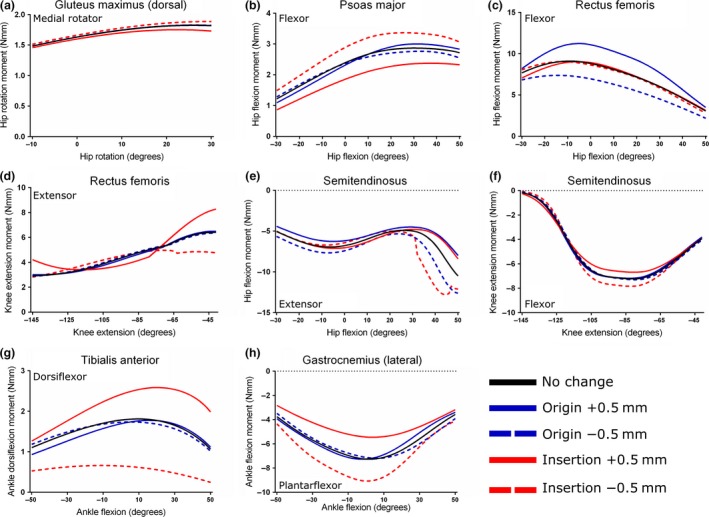
Sensitivity analysis of the Gluteus maximus (dorsal) (a), Psoas major (b), Rectus femoris (c, d), Semitendinosus (e, f), Tibialis anterior (g) and lateral Gastrocnemius (h) muscles. Here, the coordinates of origin and insertion were changed ± 0.5 mm in turn to test the effect on maximal muscle moment.

The second part of the sensitivity analysis involved sequentially moving in turn the coordinates of origin and insertion for select musculotendon units ± 0.5 mm along a defined axis to test the relative effects on maximal isometric moment vs. joint angle (Fig. 11).

Altering the insertion coordinates of GM (d) had a small but noticeable effect on peak maximal hip rotation moment (Fig. 11a). Moving the insertion on the femur 0.5 mm distally caused a 3% increase in peak moment, from 1.82 Nmm to 1.88 Nmm at 26 ^o^ of hip rotation. Moving the insertion 0.5 mm proximally caused a larger decrease in moment, to 1.74 Nmm, a 4% decrease. Moving the point of origin was found to have little effect on peak moment.

The maximal hip flexion moment of PMA was highly sensitive to changes in insertion coordinates, but relatively insensitive to similar changes in origin (Fig. 11b). Moving the insertion 0.5 mm distally along the femur resulted in a 16% increase in peak hip flexion moment, from 2.86 Nmm to 3.33 Nmm at 30 ^o^ of hip flexion, while moving it 0.5 mm proximally caused a decrease to 2.37 Nmm, a 17% decrease. Similar to the GM (d), altering the coordinates of the origin had little effect on hip flexion moment.

The peak maximal hip flexion moment of RF was more sensitive to changes in origin than insertion (Fig. 11c). Moving the origin on the anterior inferior iliac spine cranially 0.5 mm caused a 23% increase in peak moment, from 9.09 Nmm (at −9 °) to 11.23 Nmm (at −5 ^o^). Moving the origin cranially caused a 20% decrease of the peak hip flexion moment, to 7.32 Nmm (at −14 ^o^). The RF muscle's maximal knee extension moment, however, was more sensitive to changes in insertion coordinates (Fig. 11d). Moving the insertion cranially along the patella caused an increase in peak moment, from 6.41 Nmm at −41 ^o^ to 8.27 Nmm, a 30% increase. A movement of the insertion caudally resulted in a decrease in peak knee extension moment to 4.76 Nmm, a 26% decrease.

Similar to the RF muscle, changes in origin coordinates had the greatest effect on peak ST maximal hip flexion moment, with a move 0.5 mm caudally along the pelvis causing a 19% increase, from −10.60 Nmm at 50 ^o^ of hip flexion to −12.65 Nmm (Fig. 11e). Moving the origin 0.5 mm cranially resulted in a decrease of maximal hip extension moment, to −8.10 Nmm, a 24% decrease. Altering the insertion coordinates also had a noticeable effect on hip extension moment. Moving the insertion on the medial aspect of the tibia 0.5 mm distally caused the maximal hip extension moment at 50 ^o^ of hip flexion to increase to −12.12 Nmm, a 14% increase; however, the peak of hip extension moment increased 21%, to −12.82 Nmm, which occurred at 44 ^o^. The peak maximal knee extension moment of ST was most sensitive to changes in insertion coordinates, and relatively insensitive to changes in its origin (Fig. 11f). Moving the insertion distally along the tibia resulted in an 8% increase in peak knee flexion moment, from −6.86 Nmm to −7.43 Nmm at −83 ^o^ of flexion. Moving the insertion proximally caused a decrease in moment, to −6.69 Nmm, a 7% decrease. Moving the origin was found to have little effect on ST's maximal knee flexion moment.

Both the maximal peak dorsiflexion moment arm of the TA muscle, and the angle at which this peak occurs, were highly sensitive to changes in insertion coordinates (Fig. 11g). Moving the insertion 0.5 mm cranially resulted in a 43% increase in peak moment, from 1.81 Nmm at 10 ^o^ of dorsiflexion to 2.59 Nmm at 20 ^o^. Moving the insertion caudally caused a decrease of the peak moment to 0.66 Nmm at 10 ^o^, a decrease of 64%. Altering the coordinates of the origin had a negligible effect on the maximal ankle dorsiflexion moment.

Altering the insertion coordinates of the LG muscle had the greatest effect on its peak ankle plantarflexion moment, with a movement caudally resulting in a 25% increase, from −7.27 Nmm to −9.06 Nmm at −2 ^o^ (Fig. 11h). Moving the insertion cranially caused a decrease in the peak plantarflexion moment to −5.45 Nmm, a decrease of 25%. Similar to the TA muscle, moving the origin had relatively little effect on the LG's peak ankle plantarflexion moment.

## Discussion

### Moment arms

A muscle's moment arm is a measure of the effectiveness with which its contractile force can generate a rotational force, or a torque, about a joint in a given position. Estimating moment arms of the pelvic and hindlimb muscles of the mouse musculoskeletal model is therefore important in order to not only elucidate their functional roles in a variety of movements, but also to investigate the effectiveness with which they can perform these movements in particular joint positions.

As an important first step in the assessment of the robustness of the musculoskeletal model, the modelling framework was used to plot moment arm vs. joint angle for each musculotendon unit (Tables 3 and 4). This was done for each muscle group acting around the hip, knee and ankle joints through flexion–extension, adduction–abduction or medial–lateral (internal–external) rotation. These plots (Figs 5, 6, 7) concur with the initial classification of the muscles into functional groups as outlined in Tables 3 and 4, and also support the qualitative accuracy of the attachment point placement within the model.

In an analysis of cat hindlimb muscle moment arms, Young et al. (1993) noted that muscles that have moment arms whose values cross zero with a negative slope within a particular joint's range of motion could function to provide intrinsic stabilisation to that joint. Several muscles within the mouse hindlimb model possess similar zero‐crossing moment arms: OE (through hip medial–lateral rotation), M. gracilis posterior (GP; through hip adduction–abduction), PECT (through hip flexion–extension), as well as M. popliteus (POP), MG, LG, PLANT, BFP (cr) and BFP (m) (through knee flexion–extension). These ‘stabilising muscles’ are similar to those found in a rat hindlimb (Johnson et al. 2008), where PECT, OE and OI were specifically noted as having moment arms that cross zero. However, several studies have also found that this potentially self‐stabilising property is not limited to small terrestrial mammals, as evidence of self‐stabilising properties have been found in various muscles of the avian hindlimb (Hutchinson et al. 2015), as well as Mesozoic dinosaur/archosaur hind limbs (Bates & Schachner, 2012; Bates et al. 2012; Maidment et al. 2014).

As few mouse hindlimb muscle moment arm vs. joint angle data exist in the literature, select muscles’ moment arms from the mouse's hindlimb were compared with those from a rat hindlimb model (Johnson et al. 2008) in order to further inspect the robusticity of the model. While it is recognised that a rat is not simply a scaled up mouse, much of the hindlimb musculature described in architecture studies of the rat hindlimb (Eng et al. 2008; Johnson et al. 2011) is very similar to that found in the mouse (Burkholder et al. 1994; Delaurier et al. 2008; Charles et al. 2016), in terms of nomenclature, homology and architectural characteristics. Furthermore, similarities in hindlimb postures during quadrupedal locomotion between these species mean that, assuming musculoskeletal geometry is conserved, comparing moment arms between these species is valid, especially considering the absence of such data for a comparable dataset for mice. However, due to obvious differences in body size, absolute moment arm values could not be compared. Instead, moment arms from both the mouse and rat models were scaled to their respective segment length, giving dimensionless values (mm/mm), which were then plotted side‐by‐side for comparison. These plots, shown in Figs 8 and 9, show that for the majority of moment arm data tested there is good agreement between the mouse and the rat musculoskeletal models in terms of peak relative values and shape of the curves, although some noticeable differences exist. These include BFA, which shows a negative slope of its hip rotation moment arm with hip flexion in the mouse but a positive slope in the rat, and VI, which shows a positive slope of its knee extension moment arm (vs. knee flexion/extension angle) in the mouse but a negative slope in the rat. Additionally, the mouse TA has a peak ankle dorsiflexion moment arm/leg length that is less than half of that of the rat TA.

The differences in moment arms observed between the mouse and rat models could have arisen for several reasons. First, it is possible that these discrepancies could be accounted for by the different methods employed to develop the two hindlimb models. While developing their rat model, Johnson et al. (2008) used stereophotogrammetry to determine the centroids of muscle attachment points relative to bony landmarks based on three rat hindlimb specimens. In the construction of our mouse model, muscle origin and insertion coordinates were determined based on 3D bone and muscle meshes created using digital segmentation of I_2_KI enhanced micro‐CT scans of one mouse hindlimb specimen. The muscle models were then placed as close to the estimated centroid of muscle attachment as possible within the software framework. While this may seem a less precise method of determining muscle attachment points, it has been suggested that the centroid of muscle attachment may not be the centre of force generation during normal muscle contractions. This point is thought to vary *in vivo* depending on patterns of motor unit recruitment, which may place less importance on determining the exact central point of a muscle attachment when investigating muscular force generation around a joint (Monti et al. 2001; O'Neill et al. 2013).

Another possible reason for major differences between the muscle moment arms of the mouse and rat models is the use of wrapping objects or ‘via points’. These were used in the construction of the musculoskeletal model in order to prevent musculotendon units passing through other anatomical structures such as bones or other muscle models and constrain them to a biologically realistic path of action. This difference in the usage of geometric constraints on muscle paths, however, is very likely to have a large effect on muscle moment arms (O'Neill et al. 2013, also see Hutchinson et al. 2015 for discussion of counter‐examples in avian limbs). Apparently, neither wrapping objects nor ‘via points’ were used by Johnson et al. (2008) in the construction of their rat hindlimb model, which could explain some of the differences in moment arms seen between the two models, especially in the cases of BFA, VI and TA.

### Sensitivity analysis

Throughout the development of the musculoskeletal model, the architecture data of the 39 muscles of the hindlimb and pelvis were measured empirically, whereas origins and insertions were determined through observations from 3D meshes of muscles and bone. Both these methods carry a potentially large amount of observer error, so in order to determine the degree to which each measured force‐generating variable or attachment point of each muscle affected the output of the model, a sensitivity analysis was performed on a selection of hindlimb musculotendon units. This involved increasing in turn each force‐generating parameter by 5%, followed by moving the coordinates of origin and insertion ± 0.5 mm along a defined axis (Figs 10 and 11).

Altering *F*_max_ caused a substantial change in the maximal joint moment of all the muscles tested, and in some cases [e.g. GM (d), PMA, ST through knee flexion and RF] it had the greatest effect, with these muscles showing low sensitivities to changes in *L*_f_, *L*_ts_ and fibre pennation angle. As with similar sensitivity analyses (O'Neill et al. 2013), this was found to be a direct and linear effect, with +5% change in *F*_max_ causing about a 5% increase in maximal moment in all muscles tested. This is expected and not surprising given the direct relationship between *F*_max_ and maximal muscle moment (Zajac, 1989). However, given that *F*_max_ is also directly proportional to PCSA, a muscle property entirely determined by its architectural characteristics, it does reinforce the importance of accurately determining skeletal muscle architecture during the creation of a musculoskeletal model. The usage of *F*_max_ in the sensitivity analysis here also provides a baseline for comparison with the influence of other parameters with more complex sensitivity to changes as follows.

In some muscles (ST through hip flexion, TA and LG), changing the value of *F*_max_ did not have the greatest effect on maximal muscle moment. In these cases, altering *L*_ts_ caused the greatest change in muscle moment although, unlike *F*_max_, changing this parameter +5% resulted in a decrease in maximal moment of the muscle's primary function. The extent to which moment was affected by changes in *L*_ts_ appears to be influenced by the relationship between tendon length and fibre length, with muscles with long compliant tendons and *L*_ts _: *L*_f_ values > 1, such as TA and LG (2.39 and 2.54, respectively), more greatly affected by changes in *L*_ts_ relative to non‐tendinous muscles such as PMA and ST, with *L*_ts _: *L*_f_ values < 1 (0.72 and 0.32, respectively). These findings agree with those of previous sensitivity analyses of musculoskeletal models (Delp et al. 1990; Out et al. 1996; Redl et al. 2007; O'Neill et al. 2013), and confirm the presence of this relationship in small, non‐cursorial quadrupeds, extending it beyond the previously studied humans and their close relatives.

Despite the previously established importance of *L*_ts_ in musculoskeletal modelling, there is currently no method of determining this parameter experimentally or directly through cadaveric measurements. Here, a numerical optimisation procedure from Manal & Buchanan (2004) was used, which estimates *L*_ts_ based on muscle‐specific properties such as minimum and maximum musculotendon lengths and normalised fibre lengths, both obtained from the model, as well as *L*_f_ and pennation angle. This re‐emphasises the importance of accurately determining both musculoskeletal geometry and architecture throughout the process of developing a musculoskeletal model in order to give the truest estimate of *L*_ts_ possible. However, the procedure of estimating *L*_ts_ carries several inherent assumptions that may limit its muscle‐specific accuracy. The calculation assumes that: (1) fibre length was measured at optimal pennation angle (which is hard to guarantee); and (2) the muscle operates on the ascending portion of a normalised force–length curve (i.e. passive elastic force does not contribute to overall muscle force; Zajac, 1989). In practice is it hard to overcome these limitations, so relying on estimates of *L*_ts_ may, for the foreseeable future, inherently reduce the reliability of the musculoskeletal modelling process.

As mentioned above, *L*_ts_ is not a variable that is normally measurable, but is used in modelling to give an indication of the in‐series elasticity of a muscle‐tendon unit, and takes into account any potential external or internal tendon (e.g. aponeurosis) within a whole musculotendon unit, as defined by the generic Hill‐type muscle model used here (Fig. 2a). Typically, when measuring tendon lengths of muscles in architecture studies, only the external tendon is measured. In reality, however, many muscles, particularly those with large fibre pennation angles, have substantial internal tendons (Fig. 2b), which are thought to be as important in determining a muscle's contraction dynamics as external tendons (Rack & Westbury, 1984; Proske & Morgan, 1987; Zajac, 1989). The values of *L*_ts_ used to define the contraction dynamics of the muscles within this model were compared with external tendon length (*L*_t_) values for the same muscles measured from dissection experiments (from Charles et al. 2016) in order to assess the relationship between these two variables (Table 9), which is very seldom done in such modelling studies. This was only possible for some muscles (mostly the distal muscles of the hindlimb), as those of more proximal functional groups had little or no measurable external tendon. The *L*_ts _: *L*_t_ values for the distal hindlimb muscles (plus the quadriceps femoris group: RF, VL, VM and VI) show that *L*_ts_ is generally much larger than *L*_t_, which is expected given the definition of *L*_ts_ (Zajac, 1989). However, this varies considerably between the muscles, with PLANT having the highest value (*L*_ts _: *L*_t _= 3.61) and M. peroneus digiti quinti (PDQI) the lowest (*L*_ts _: *L*_t _= 1.25). This indicates a fundamental architectural difference between these muscles that is consistent with their gross anatomy. Muscles with higher *L*_ts _: *L*_t_ values (> 2) are likely to have a larger component of their entire tendon located within the muscle belly rather than as an external tendon, whereas muscles with lower values (< 2) likely have a relatively large external tendon(s) and little internal aponeurosis. This variation could relate to general function, with ‘swing phase’ muscles, such as TA, EDL and the peroneus muscles of the lateral compartment of the leg having longer external tendons than more powerful ‘stance phase’ (antigravity) muscles, such as the ‘triceps surae’ group (MG, LG and PLANT) and the quadriceps femoris group (RF, VM, VL and VI). Unfortunately, this hypothesis is not immediately testable, but could give some insight into the architectural and functional significance of *L*_ts_, which is generally regarded as an abstract concept unique to musculoskeletal models.

**Table 9:** The relationship between tendon slack length (*L*_ts_) and measured external tendon length (*L*_t_) for 18 distal musculotendon units included in the musculoskeletal model, given as the ratio *L*_ts _: *L*_t_. *L*_ts_ was estimated using a numerical optimisation procedure from Manal & Buchanan (2004)

Musculotendon unit	Abbreviation	Groups	*L*_ts_ (m)	*L*_t_ (m)	*L*_ts _: *L*_t_
Rectus femoris	RF	Knee extensors	0.00853	0.00355	2.40
Vastus medialis	VM	Knee extensors	0.00768	0.00355	2.16
Vastus lateralis	VL	Knee extensors	0.00735	0.00355	2.07
Vastus intermedius	VI	Knee extensors	0.00702	0.00355	1.98
Tibialis anterior	TA	Ankle dorsiflexors	0.01180	0.00610	1.93
Extensor digitorum longus	EDL	Ankle dorsiflexors	0.02378	0.01459	1.63
Extensor hallucis longus	EHL	Ankle dorsiflexors	0.01793	0.01082	1.66
Madial gastrocnemius	MG	Ankle plantarflexors, knee flexors	0.01395	0.00497	2.80
Lateral gastrocnemius	LG	Ankle plantarflexors, knee flexors	0.01389	0.00434	3.20
Soleus	SOL	Ankle plantarflexors	0.00740	0.00304	2.44
Plantaris	PLANT	Ankle plantarflexors	0.01517	0.00420	3.61
Flexor digitorum longus	FDL	Ankle plantarflexors	0.02761	0.01512	1.83
Tibialis posterior	TP	Ankle plantarflexors	0.01500	0.00611	2.46
Peroneus longus	PL	Ankle everters, ankle plantarflexors	0.01408	0.00794	1.77
Peroneus tertius	PT	Ankle everters, ankle plantarflexors	0.01122	0.00645	1.74
Peroneus brevis	PB	Ankle everters, ankle plantarflexors	0.01005	0.00677	1.48
Peroneus digit quarti	PDQA	Ankle everters, ankle plantarflexors	0.02357	0.01265	1.86
Peroneus digiti quinti	PDQI	Ankle everters, ankle plantarflexors	0.01959	0.01569	1.25

*L*_t_ values were taken from previously measured skeletal muscle architecture (Charles et al. 2016). See Fig. 2 for graphical consideration.

As the second part of the sensitivity analysis of the mouse hindlimb model, the coordinates of each muscle's origin or insertion were altered in turn to test the effect on maximal moment‐generating capacity of each muscle. The joint that was being acted upon or the movement being modelled had a large influence on whether moving the origin or the insertion had a greater effect on muscle moment. For the majority of muscles and joint rotations tested, altering the coordinates of insertion had the greatest effect on moment, rather than origin.

This, unsurprisingly but importantly, was found to be the case for the muscles in which the point of insertion was closer to the centre of joint rotation than the origin. For bi‐articular muscles such as the ST and RF, both of which act around the hip and the knee, changing the coordinates of origin had a greater effect on hip flexion–extension moment, as the origins were closer to the hip joint than the insertions. Furthermore, a movement of an attachment point away from the centre of joint rotation resulted in an increase in moment, while a movement towards the joint caused an opposite effect. This is of course unsurprising, as a greater distance between an attachment point and a joint's rotational centre would increase the musculotendon units’ moment arm, and therefore moment, around that particular joint. This sensitivity of muscle moment arm to changes in muscle attachment points has been reported previously, such as in musculoskeletal models of the human elbow joint (Murray et al. 1995) and the chimpanzee hindlimb and pelvis (O'Neill et al. 2013). Given the high sensitivity of most of the muscles tested to insertion coordinates, it is fortunate therefore that points of insertion, especially for muscles with long tendons, tend to be better defined in mice than points of origin, which are generally much broader. Despite the current findings for this aspect of the sensitivity analysis, its net effect should be to bolster confidence in the qualitative, if not quantitative, accuracy and reliability of musculoskeletal models.

When building a musculoskeletal model, it is desirable to determine muscle attachments to the highest degree of accuracy possible, as even small movements of either an origin or insertion coordinate can potentially lead to a large change in the model output. However, the degree to which this change is affected is highly variable and depends on several other factors noted above.

## Conclusions

Here the methods used to construct a 3D musculoskeletal model of a mouse's hindlimb and pelvis were presented using the biomechanical software framework (Zajac, 1989; Delp & Loan, 1995, 2000). The model, consisting of 7 degrees of joint freedom and 44 musculotendon units in total, was constructed based on previously gathered muscle architecture and musculoskeletal geometry data. Plots of muscle moment arm vs. joint angle justified the muscles’ functional group placements, and support the general reliability of the model and placement of the musculotendon units. As a further assessment of the model reliability, muscle moment arms were compared with those from a musculoskeletal model of a similar non‐cursorial rodent, the rat, developed by Johnson et al. (2008). Due to body size differences, the moment arms were normalised to their respective segment lengths to facilitate accurate comparisons. In most cases, the shape and relative magnitudes of the moment arms were found to be similar, further supporting the mouse hindlimb model. However, some differences were found, which most likely reflects the different methods used to construct the two models. Given the strong similarities in musculoskeletal anatomy rats and mice (Burkholder et al. 1994; Delaurier et al. 2008; Eng et al. 2008; Johnson et al. 2011; Charles et al. 2016), this highlights how the methods used to create a musculoskeletal model can strongly influence the results obtained from it (Hutchinson et al. 2015).

The results of the sensitivity analyses for several mouse hindlimb muscles show that there are many factors that can have a large effect on the output of a musculoskeletal model, which need to be carefully considered when building such a model. Changes in *L*_ts_ were found to cause the greatest changes in maximal muscle moment in most cases, although this (as expected on theoretical grounds; e.g. Zajac, 1989) was seen most in the more distal, tendinous muscles of the hindlimb. Changing the coordinates of muscle attachment points was also found to have substantial effects on maximal muscle moments, with movements of insertion locations causing larger changes in moment in the majority of muscles tested.

This study represents crucial initial steps in the creation of a dynamic musculoskeletal model of the mouse hindlimb and pelvis, into which future implementations can incorporate other data, such as mouse hindlimb kinematics (Hutchinson et al. 2015) as well as ground reaction force and electromyography data, to more fully simulate a moving mouse hindlimb.

A major aim of the current study was to contribute a model that has broad utility. The model presented here would facilitate studies in normal and pathological neuromuscular physiology, and the genetic bases of, and gene therapies for, further diseases. Finally, the current model enables novel comparative and detailed mechanistic work in locomotor neuromechanics. In this last area, genetically targeted tools such as optogenetics are beginning to make it possible to isolate and specifically manipulate sensory and motor pathways, for example. Viral methods make it possible to target specific muscles for activation (Towne et al. 2013), and for modulation of sensory feedback (Iyer et al. 2014). With improvement of genetic targeting ability, it is likely that it would be possible to target specific sense organ classes, for example. Gaining scientific insight from such experimental work will benefit from, if not require, a carefully created musculoskeletal model. These and other potential future usages are strong justification for rigorous design and sensitivity analysis of musculoskeletal models of various species and organ systems in general.

## Author contributions

Conceived study: JRH, AJS, DJW; acquired data: JPC, OC; data analysis/interpretation: JPC, JRH; drafted the manuscript: JPC, JRH, DJW, OC; critically revised the manuscript and approved the final draft: JPC, JRH, DJW, OC, AJS.

## Supporting information


**Fig. S1**. Sensitivity analysis of selected mouse hindlimb muscles, in which maximum isometric force (*F*_max_), muscle fibre length (*L*_f_), tendon slack length (*L*_ts_) and fibre pennation angle were increased by 1 standard deviation of the mean value in turn to test the effect on maximal muscle moment.
