# Supplementary figures and images for: Muscle moment arms and sensitivity analysis of a mouse hindlimb musculoskeletal model
Source: J Anat. 2016 May 12;229(4):514–35. doi: 10.1111/joa.12461 (PMC5013061; doi:10.1111/joa.12461)

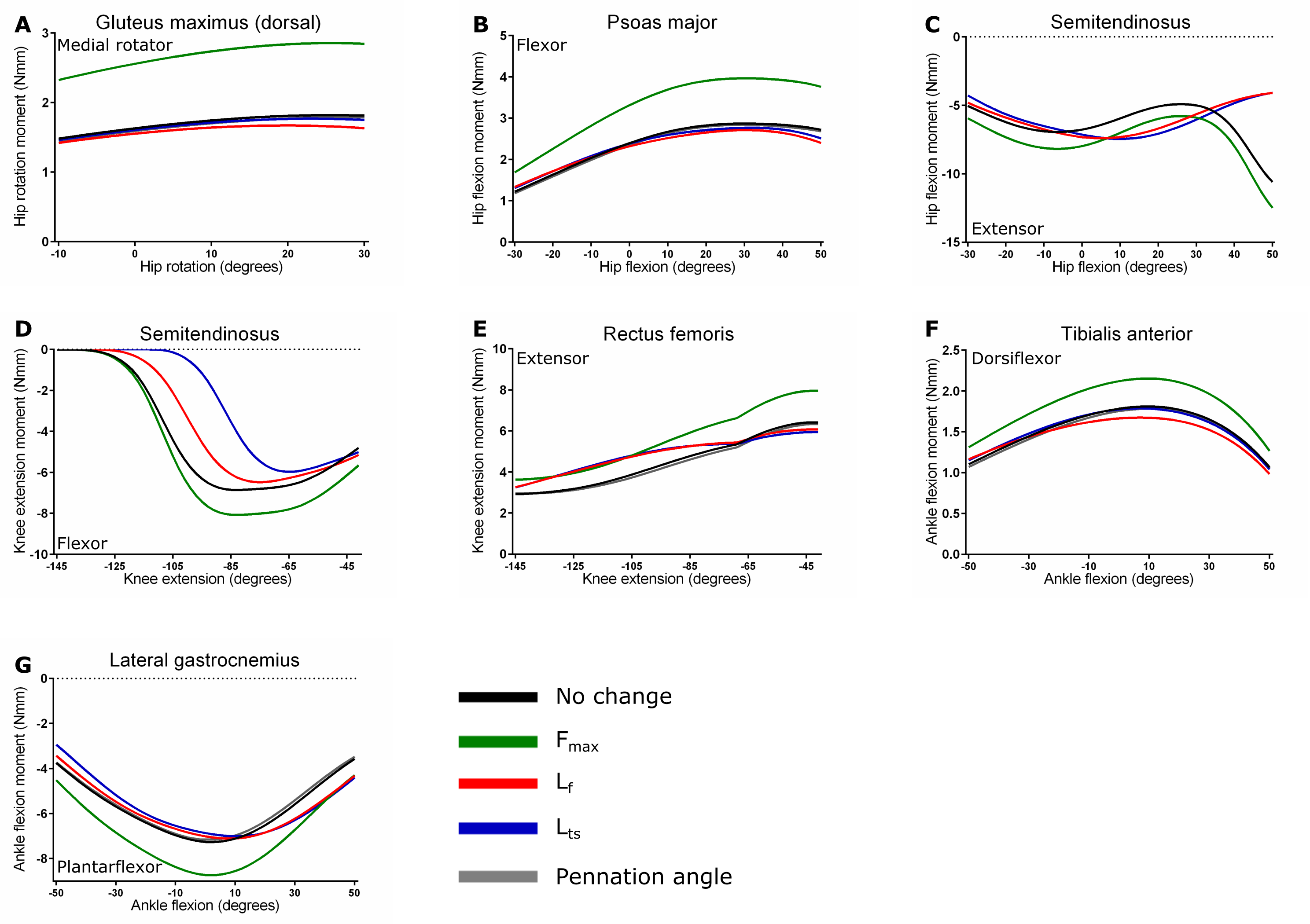

Supplement: Supplementary file 1 — Fig. S1. Sensitivity analysis of selected mouse hindlimb muscles, in which maximum isometric force (F max), muscle fibre length (L f), tendon slack length (L ts) and fibre pennation angle were increased by 1 standard deviation of the mean value in turn to test the effect on maximal muscle moment. [file JOA-229-514-s001.tif]
